# Enriching Proteolysis Targeting Chimeras with a Second Modality: When Two Are Better Than One

**DOI:** 10.1021/acs.jmedchem.2c00302

**Published:** 2022-07-11

**Authors:** Alessandra Salerno, Francesca Seghetti, Jessica Caciolla, Elisa Uliassi, Eleonora Testi, Melissa Guardigni, Marinella Roberti, Andrea Milelli, Maria Laura Bolognesi

**Affiliations:** † Department of Pharmacy and Biotechnology, Alma Mater Studiorum - University of Bologna, Via Belmeloro 6, 40126 Bologna, Italy; ‡ Department for Life Quality Studies, Alma Mater Studiorum - University of Bologna, Corso d’Augusto 237, 47921 Rimini, Italy

## Abstract

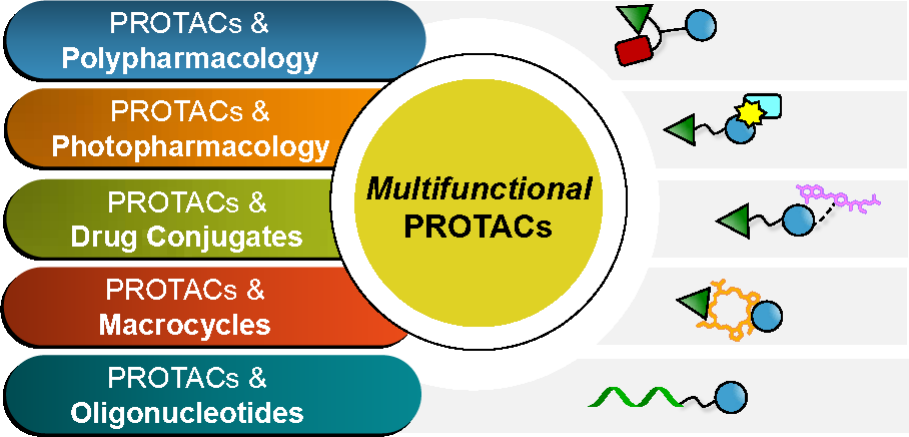

Proteolysis targeting chimera (PROTAC)-mediated protein degradation has prompted a radical rethink and is at a crucial stage in driving a drug discovery transition. To fully harness the potential of this technology, a growing paradigm toward enriching PROTACs with other therapeutic modalities has been proposed. Could researchers successfully combine two modalities to yield *multifunctional* PROTACs with an expanded profile? In this Perspective, we try to answer this question. We discuss how this possibility encompasses different approaches, leading to *multitarget* PROTACs, *light-controllable* PROTACs, PROTAC *conjugates*, and *macrocycle-* and *oligonucleotide-based* PROTACs. This possibility promises to further enhance PROTAC efficacy and selectivity, minimize side effects, and hit undruggable targets. While PROTACs have reached the clinical investigation stage, additional steps must be taken toward the translational development of *multifunctional* PROTACs. A deeper and detailed understanding of the most critical challenges is required to fully exploit these opportunities and decisively enrich the PROTAC toolbox.

## Introduction

The use of small molecules for protein target modulation is the classic drug discovery approach.^1^ Broadly defined as chemical compounds with a low molecular weight (MW = 0.1–1 kDa),^2^ small molecules have both advantages and disadvantages. They generally bind to the protein of interest (POI)—enzymes, ion channels,, or receptors typically endowed with a well-defined ligand-binding site—and modulate its function.^3^ Small molecules engage their targets by various mechanisms of action (MoAs) and, depending on their localization, can act both intracellularly and extracellularly. However, several proteins lack binding sites and catalytic activity, or have catalytic-independent functions, making their modulation difficult to achieve. Consequently, more than 80% of proteins are considered “undruggable”.^4^ This percentage comprises critical targets such as transcription factors (TFs), scaffolding proteins, or non-enzymatic proteins inside the cells.^5^

Over the years, alternative therapeutic approaches have been developed to face these challenges.^6^ They include monoclonal antibodies (mAbs),^7^ antisense oligonucleotides (ASOs),^8^ small-interfering RNAs (siRNAs),^9^ CAR T-cell therapies,^10^ and, more recently, CRISPR-Cas9 technology.^11^ However, their development has faced many problems that still today limit their clinical applicability.

Notwithstanding a subtle perception that they have run their course, small molecules continue to be the mainstay of the pharmaceutical research. In 2021, the U.S. Food and Drug Administration (FDA) approved 50 new molecular entities—just 3 fewer than in 2020, and the third highest total in the past 20 years. Among these, 31 (62% of new approved therapeutics) were small molecules, confirming their critical role in the drug pipeline.^12^

Certainly, the small-molecule discovery field has been revitalized by the emergence of a truly revolutionary modality based on PROteolysis TArgeting Chimeras (PROTACs).

This ground-breaking approach uses small molecules, i.e., PROTACs, to control protein levels rather than modulate their function.^13−15^ PROTACs do not inhibit the POI, but they induce its removal by binding and harnessing the cell disposal ubiquitin–proteasome system (UPS). Such PROTAC-mediated protein degradation (P-mPD) offers an extraordinary strategy to enhance classic drug discovery approaches, giving the opportunity to target the “undruggable” proteome.^5^

**Figure 1:**
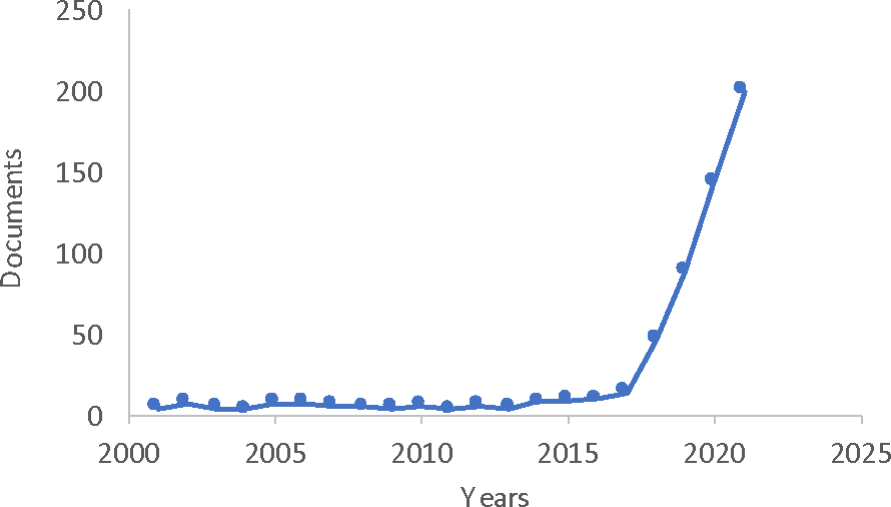
Number of articles per year featuring “proteolysis targeting chimera” and/or “PROTAC” in the title, abstract, or keywords (Scopus search, January 2022). Reviews, book chapters, editorials, and conference papers are not included.

**Figure 2:**
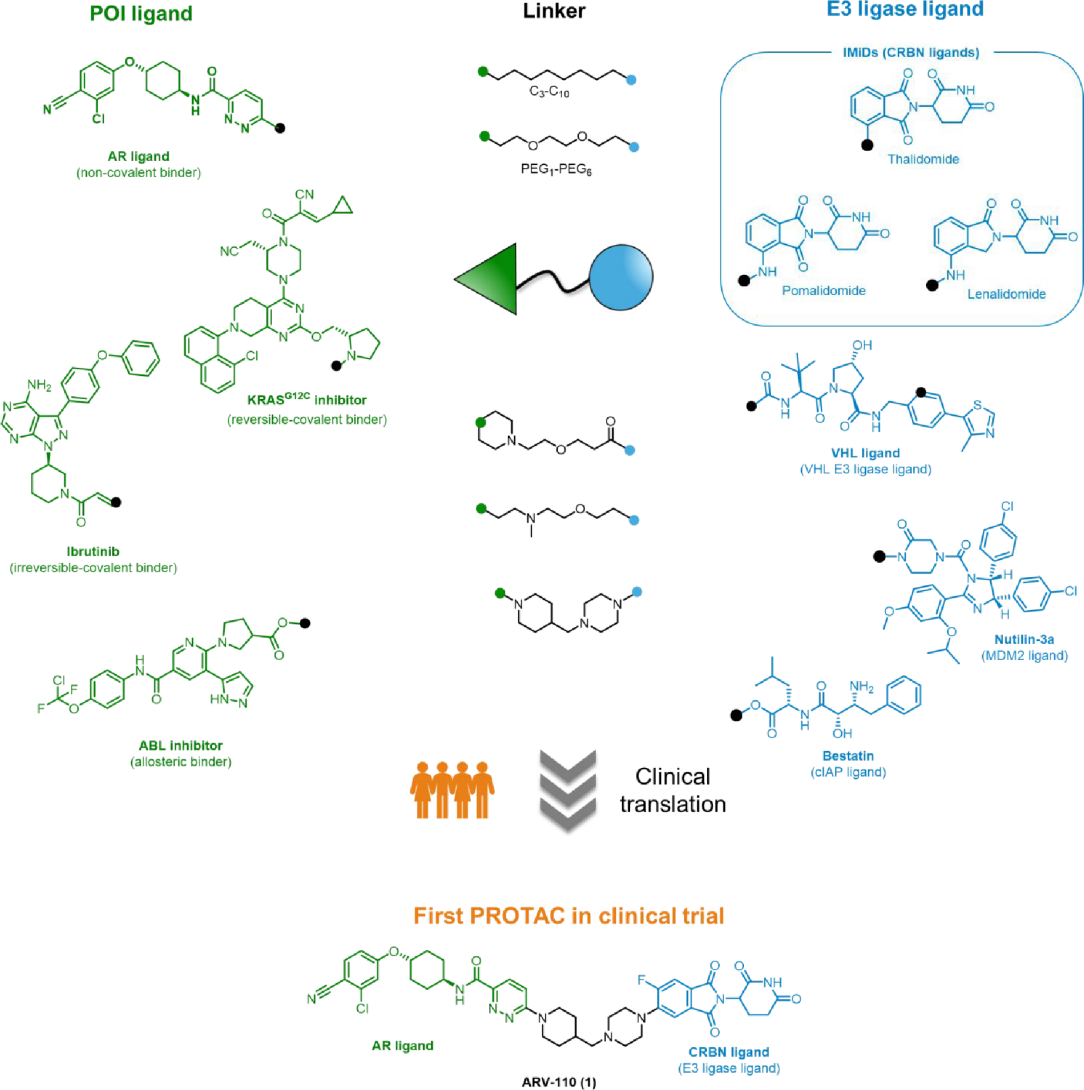
Elements of PROTAC design: the judicious combination of the proper *POI ligand*, *linker*, and *E3 ligase ligand* has led to the clinical investigation of ARV-110 (**1**).

The first report about P-mPD was published in 2001 by the groups of Craig M. Crews and Raymond J. Deshaies,^16^ but it remained largely under-explored until 2015. Since then, PROTACs have gained formidable attention from both academia and pharmaceutical/biotechnology companies.^17,18^ A Scopus search (January 2022) for articles containing “Proteolysis Targeting Chimera” or “PROTAC” in the title, abstract, or keywords retrieved, starting from 2001, the impressive number of 590 entries (Figure 1). Remarkably, 490 (83%) of these publications are dated between 2017 and 2021 and appear in high-impact medicinal chemistry/chemical biology journals, e.g., *Journal of Medicinal Chemistry* (59), *Journal of the American Chemical Society* (17), *ACS Chemical Biology* (19), and *ACS Medicinal Chemistry Letters* (18). The explosion observed from 2017 onward is likely related to a shift from peptide-based to fully synthetic small-molecule degraders, which are more promising in terms of drug-like property optimization and oral bioavailability.^19^ In this respect, a breakthrough was the discovery of the immunomodulatory imide drug (IMiD) thalidomide (see Figure 2 in section 1) as a cereblon (CRBN) E3 ligase ligand.^20^

To further confirm the success and the amazing potential of the approach, in 2019, Arvinas Therapeutics began the first ever clinical study of a targeted protein degrader, ARV-110 (**1** in Figure 2), an orally bioavailable PROTAC for the potential treatment of metastatic castration-resistant prostate cancer, which is now in Phase II clinical trial.^21^ As if a new golden goose has been found, all the major players in drug discovery have started their own targeted protein degradation programs and brought several degraders into clinical trials.^18,22^

The medicinal chemistry behind P-mPD has grown exponentially in the past few years (as reviewed in refs (23−25)). In a recent article,^26^ Craig Crews uses the Shakespeare quotation “the past is prologue” to elegantly describe where we have been and where we are going in the field. This expression reminds us that everything that has been developed so far has occurred to prepare us for that will follow. We feel that the field has matured so much that it has already incorporated the latest developments in terms of novel medicinal chemistry strategies and novel types of drugs/chemotypes, collectively highlighted in a recent Editorial as “new modalities”.^27^ In other words, our interpretation is that, to overcome some of the current hurdles, the PROTAC toolbox has been expanded through the development of PROTACs endowed with a second modality, besides the specific proteasome-mediated one. The incorporation of a molecular framework, responsible for the extra modality, has provided what we envisage as “*multifunctional PROTACs*”.

In this view and to avoid unnecessary overlap with the prolific recent literature,^23,24,26,28^ the aim of this Perspective is to critically analyze the development of such *multifunctional* PROTACs. Notwithstanding their potential advantages, the reader should be aware of the intrinsic strategic risk, considering that PROTACs are “unprecedented” drugs, and the additional layer of complexity in terms of drug discovery and development. Thus, after briefly introducing the essential of PROTAC medicinal chemistry and pharmacology, we will highlight and discuss selected PROTAC case studies from this perspective.

## 1 The Essential Medicinal Chemistry of PROTACs

From a medicinal chemistry point of view, PROTACs are heterobifunctional molecules consisting of a ligand that binds the POI connected via a linker to a recruitment moiety for an E3 ubiquitin ligase (Figure 2).^29^ This has been exhaustively discussed elsewhere.^26^

Regarding the “POI ligand”, a vast array of warheads has been reported.^30^ It spans from non-covalent, irreversible, and reversible covalent ligands to allosteric ones.^31^ They are directed to more than 100 targets, including chromatin readers such as BRD4, cytoplasmic hormone receptors (e.g., AR and ER) scaffolding and regulatory proteins, aggregation-prone misfolding proteins (e.g., Tau), fusion proteins (e.g., BCR-ABL), and receptor tyrosine kinases (e.g., EGFR, HER3, FLT3).

The chemical structures of POI ligands, along with biological activities and physicochemical properties, have been collected in the PROTAC-DB database and PROTACpedia,^32^ useful resources for PROTAC practitioners.

A critical role is also played by the “linker”, featuring structurally simple alkyl or polyethylene glycol (PEG) chains, up to more rigid piperazine/piperidine-based linkers. Its length and chemical composition have been shown to impact, among others, PROTAC’s rigidity, hydrophobicity, and solubility. It is also well supported that such linker features are very important for productive ternary complex formation, degradation activity, and target selectivity.^24^ To date, linker structure–activity relationship (SAR) studies are largely empirical, and linker design still represents a bottleneck.^24^ However, recent advances in computational approaches modeling PROTAC-mediated ternary complexes could inform rational structure-based optimization.^33^

Similarly, the choice of the “E3 ligase ligand”, which can act reversibly or irreversibly, is critical for the final success. More than 600 E3 ligases are predicted to be encoded by the human genome,^34^ each with its own specificities, but only few of them have been successfully harnessed for PROTACs: e.g., mouse double minute 2 homologue (MDM2), von Hippel Lindau (VHL), Cereblon (CRBN), and cellular inhibitor of apoptosis protein (cIAP). This is mostly due to the availability of small-molecule ligands to these E3 ligases, which include, but are not limited to, those depicted in Figure 2. A comprehensive discussion of traditional and new E3 ligase ligands can be found elsewhere.^35^ It has been also demonstrated that different degradation and tissue-selective profiles are possible, depending on the recruited ligase.^36^

## 2 The Essential Pharmacology of PROTACs

**Figure 3:**
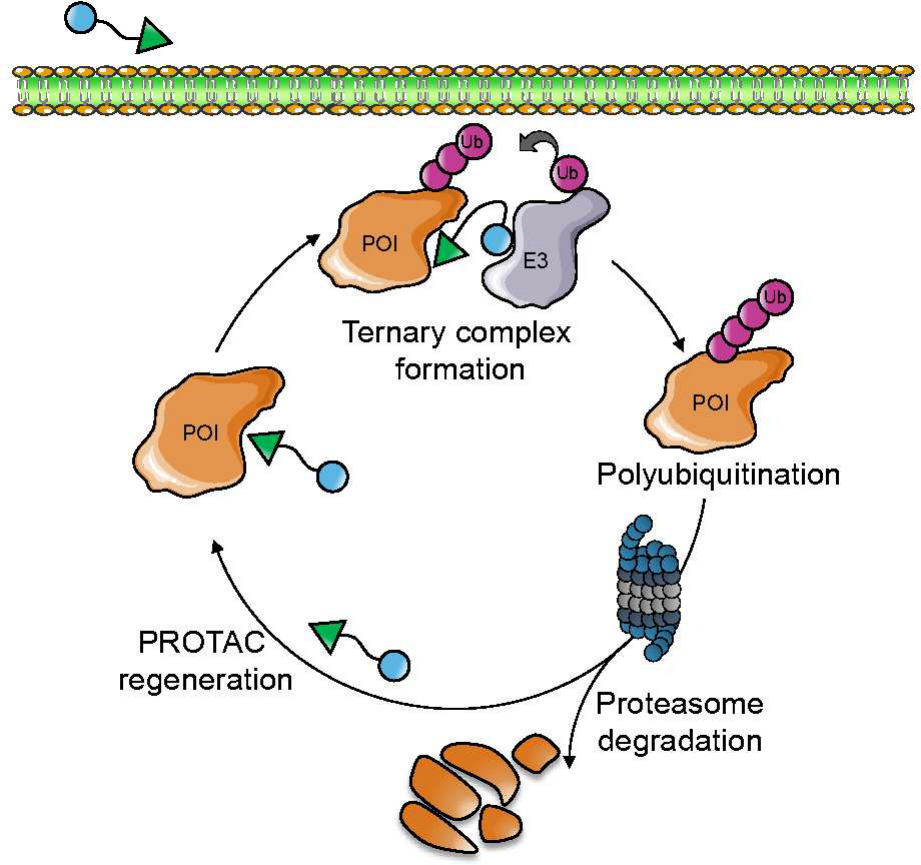
Schematic PROTAC-mediated protein degradation (P-mPD). PROTAC induces the formation of a ternary complex between the POI and an E3 ligase, bringing them in spatial proximity. POI is then polyubiquitinated, finally leading to POI degradation via the UPS.

PROTACs initiate the degradation cascade by recruiting the POI and forming a ternary complex with the E3 ligase (Figure 3). The induced proximity between the POI and the E3 ubiquitin ligase elicits ectopic ubiquitination of lysine residues of the POI surface. The ubiquitinated POI is finally recognized and degraded by the 26S proteasome.

PROTACs offer several advantages compared to “classical” small-molecule-based drugs. Indeed, protein degradation is an *event-driven* rather than *occupancy-driven* pharmacology. In this view, PROTACs catalytically remove sub-stoichiometric quantities of proteins through multiple rounds of activity and trigger potent effects even at low doses. Evidence for the catalytic nature of PROTACs has been originally provided by determining the kinetics and stoichiometry of PROTAC-induced POI ubiquitination in *in vitro* assays.^37^ However, not all the published PROTAC studies report on this aspect. On the contrary, classic small-molecules-mediated pharmacology is often achieved with >90% of target engagement;^38^ therefore, occupation of the binding site requires high drug exposure and consequently the use of a high dose, which can potentially lead to toxic on- and off-target effects. In addition, PROTACs can better circumvent some inhibitor resistance mechanisms typical of cancer and infectious diseases, including (i) point mutations, (ii) gain of scaffolding function, and (iii) target protein overexpression. This is mainly due to their event-driven pharmacology, resulting in catalytic removal of POI in its entirety and in degradation driven by binding rather than function disruption.^39^ However, acquired resistance to PROTACs by genomic alterations of E3 ligase complex core components cannot be ruled out.^40^ Moreover, and more importantly, PROTACs can expand the number of “druggable” targets since they have been demonstrated to degrade proteins lacking a catalytic site or a small-molecule binding site. This is the case, for example, of aberrant Tau in frontotemporal dementia, which is conventionally considered an intractable target because the lack of a well-defined active site.^41^

Apart from the advantages discussed above, there is still room for improvement in some areas,^42^ such as the following:

(a) Twelve PROTACs have recently reached clinical phases, some of them as therapeutic combinations with other agents to exploit synergistic effects. As a further step, the development of PROTAC-mediated dual degradation of networked proteins is promising. Although in its infancy, it may represent an effective strategy in the frame of drug-resistant or multifactorial diseases.
(b) PROTACs are not always selective and could induce degradation of other proteins (off-target effect) or unselective degradation of POIs in an undesired tissue (on-target effect). As an example, CRBN E3 ligase ligands induce degradation of some TFs (e.g., SALL4)^43^ and Ikaros family of zinc finger proteins (i.e., IKZF1 and IKZF3) by acting as molecular glue degraders.^44^ Thus, PROTACs might benefit from prodrug approaches and a spatiotemporal control.
(c) In spite of properties lying outside the classic “rule-of-five” space,^45^ when orally administered, PROTACs have been shown to induce POI degradation in any reachable cells, without differentiating between healthy and diseased cells. This has been partly overcome with the development of topical PROTACs, which avoid systemic exposure and side effects. A recent example is the androgen receptor (AR)-PROTAC GT20029 (undisclosed structure), which entered Phase I clinical trial in China for androgenetic alopecia and acne.^46^ However, when systemic administration is required, targeted delivery systems may overcome limitations of poor selectivity and *in vivo* pharmacokinetic (PK) profiles.
(d) PROTACs’ flexibility is a crucial parameter to be considered during design and development. In addition to influencing PK properties, flexibility plays an important role in influencing the ternary complex formation. In this regard, locking the linker or POI ligand into the bioactive conformation by macrocyclization strategies may facilitate ternary complex formation and enhance the PROTAC’s degradation profile.
(e) Over the years, the spectrum of targets that can be degraded by PROTACs has greatly expanded. However, some targets are still difficult to tackle via small-molecule degraders.^26^ Oligonucleotide-based therapeutics have received ever-increasing attention for their potential to modulate targets lacking hydrophobic pockets and well-defined binding sites.

Indeed, the limitations described above have been already overcome by the development of brand-new tools, recently highlighted as “novel” PROTACs, to distinguish them from “classical” PROTACs.^28^ To a closer look, we envisage that in all these cases the degradation technology has been combined with a second modality, expanding the original MoA.

**Figure 4:**
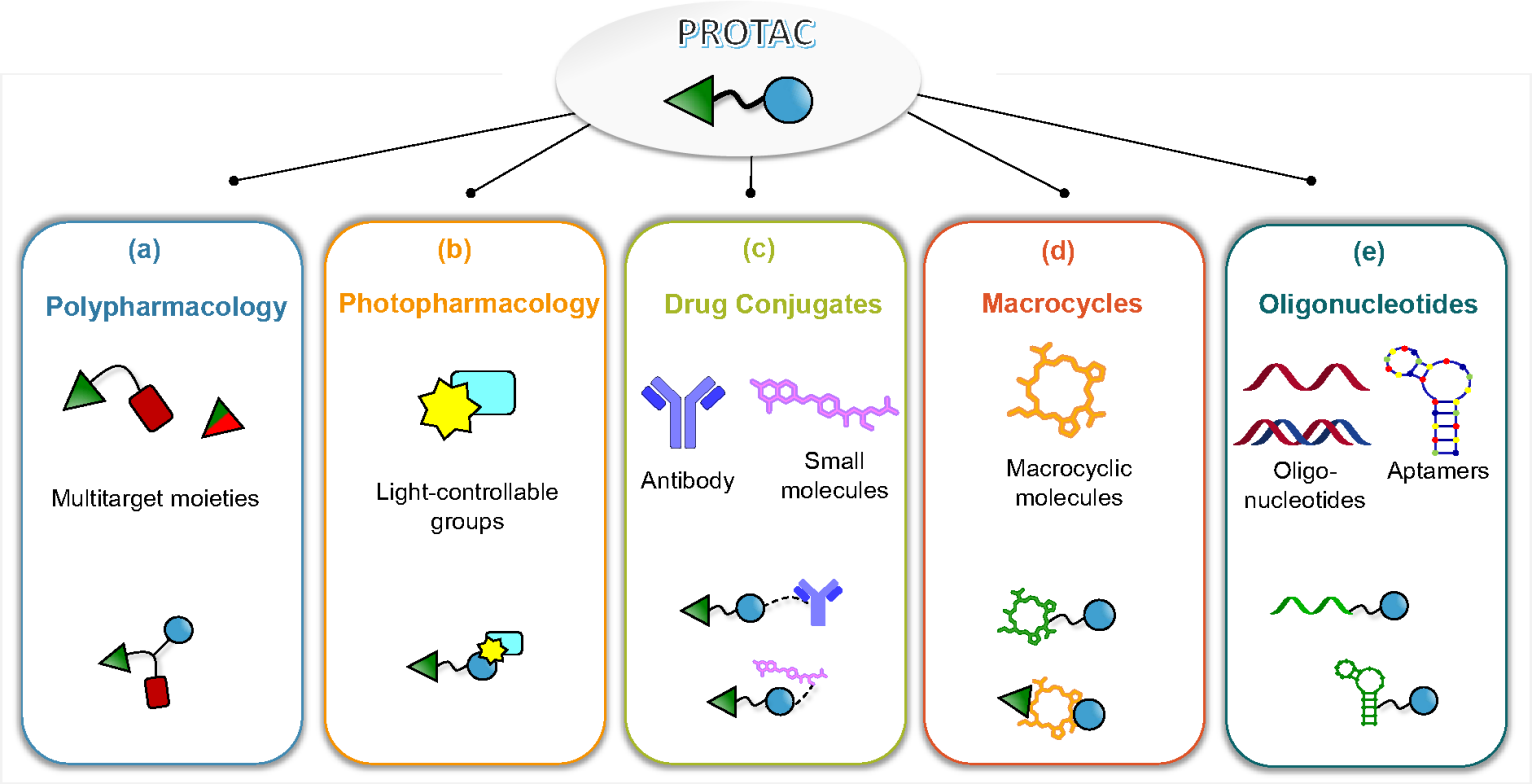
Cartoon representation of the five types of *multifunctional* PROTACs, obtained from combining PROTAC modality and (a) polypharmacology, (b) photopharmacology, (c) drug conjugates, (d) macrocycles, and (e) oligonucleotides.

From this angle, in section 3 (Figure 4a), we will discuss how PROTACs and polypharmacology have been combined in what we dub *multitarget* PROTACs, with a pronounced conceptual similarity to multitarget-directed ligands (MTDLs).^47^ In section 4 (Figure 4b), we will critically review *light-controllable* PROTACs, resulting from the combination of PROTAC technology with photopharmacology.^48^Section 5 (Figure 4c) reports on *antibody-* or *small-molecule-PROTAC conjugates* aimed at combining targeted delivery^49^ and PROTACs toward a higher selectivity, reduced toxicity, and improved PK profiles. Section 6 (Figure 4d) highlights PROTACs integrating a novel chemotype, which bears great promise in pharmaceutical discovery, i.e., macrocyclic structures.^50,51^ Finally, in section 7 (Figure 4e), we will describe how the field of PROTACs has incorporated oligonucleotide-based approaches,^52^ hence opening up exciting new avenues.

## 3 Combining PROTACs and Polypharmacology Modalities

In the era of network pharmacology,^53^ complex diseases, such as cancers and neurodegenerative diseases, are viewed as the result of a systemic breakdown of physiological networks. Given the robustness and redundancy of such diseased networks, it is unlikely that a single intervention (i.e., single-target drugs) can restore the perturbed situation. Conversely, the simultaneous modulation of several targets may contribute to achieve the desired therapeutic effect. Polypharmacology, which embodies the use of pharmaceutical agents acting on multiple targets, seems to be the best way to restore the complex diseased network.^54^ Since the term was coined in 2008,^47^ MTDLs have become a milestone in the modern medicinal chemistry and one of the most explored polypharmacological strategies. MTDLs are meant as single molecules with a potential multifaced MoA developed by framework combination of parent scaffolds.^55^ The feasibility of this approach is supported by the armamentarium of investigational and approved drugs with a multitarget profile. Among them, numerous dual inhibitors of cyclin-dependent kinases 4 and 6 (e.g., palbociclib) and serotonin–dopamine activity modulators (e.g., aripiprazole) were approved for the treatment of complex cancer and psychiatric disorders, respectively.^56^

**Figure 5:**
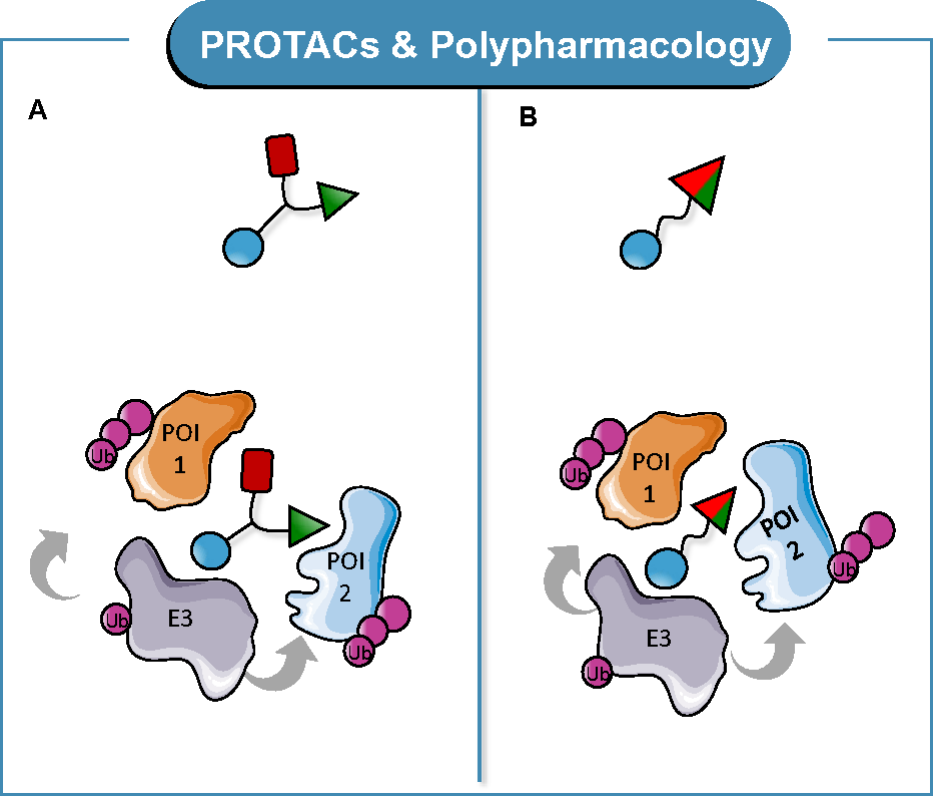
General structure and MoA of *multitarget* PROTACs: (A) two different POI ligands binding to two different POIs and (B) one dual-targeting POI ligand binding to two different POIs.

A polypharmacology profile, in terms of multiple and concerted pharmacological modulation of two or more targets, could be a suitable opportunity also for PROTACs. In this section, we report examples of *multitarget* PROTACs, referring to those that are endowed with (i) two POI ligands (Figure 5A) or (ii) a dual-targeting POI ligand (Figure 5B). The recruitment and degradation of multiple targets motivated our definition of *multitarget* PROTACs.

To note, so-called *trivalent* PROTACs^57^ have not been deliberately included, as they exploit multivalency concepts (i.e., enhanced avidity, potency, or selectivity) and not polypharmacology ones.^58^ Indeed, they embody two POI ligands that can simultaneously bind to two sites or two units of the same protein (and not to two different targets).

**Figure 6:**
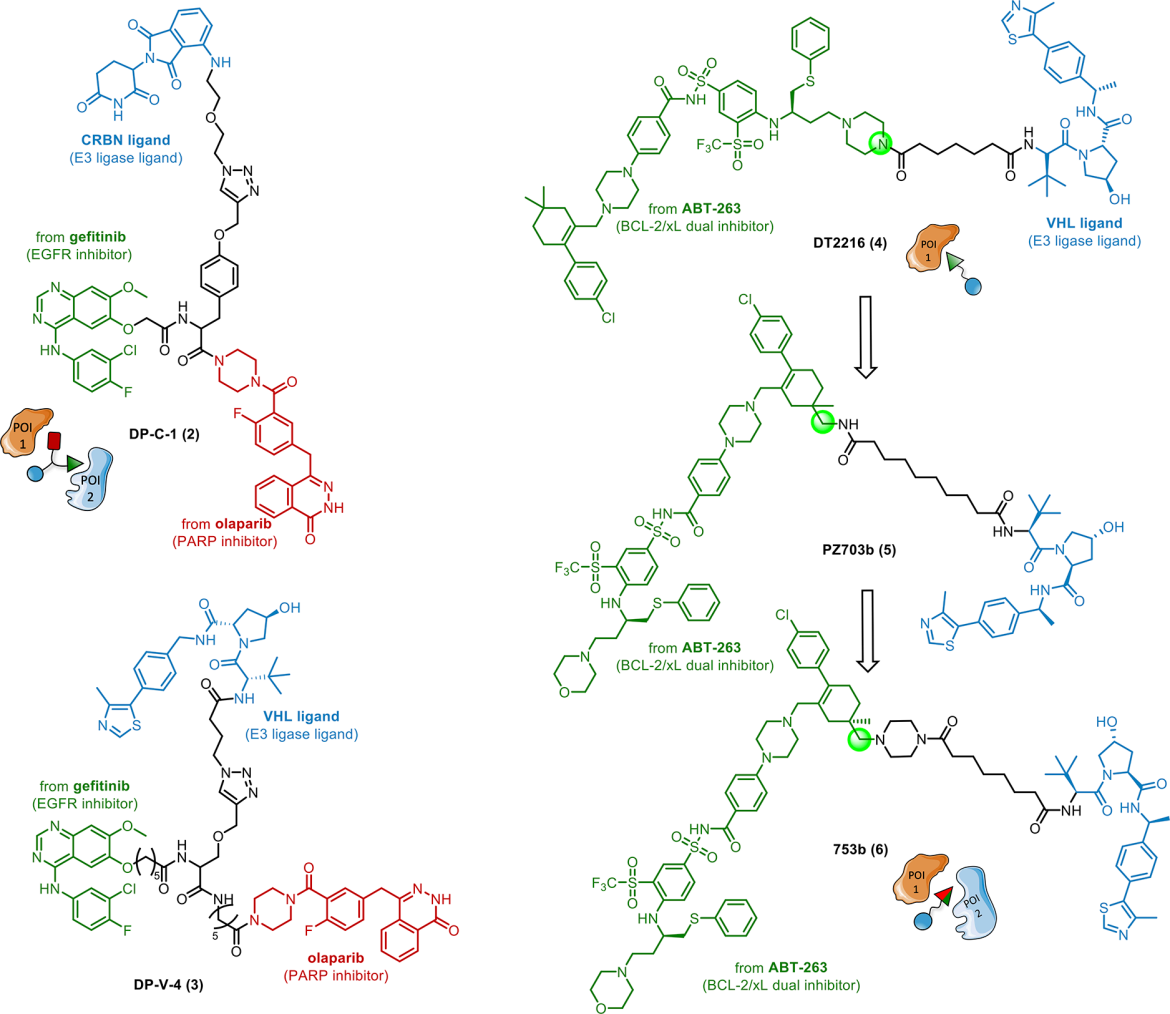
Design of *multitarget* PROTACs. For completeness, the parent compounds from which the POI ligands are derived, are indicated in green.

A library of rationally designed *multitarget* PROTACs (exemplified by case (a) in Figure 5A) has been recently reported.^59^ Particularly, gefitinib and olaparib were combined in CRBN-based or VHL-based PROTACs, with the intent to degrade two targets interconnected in cancer evolution pathways, i.e., the epidermal growth factor receptor (EGFR) and poly(ADP-ribose) polymerase (PARP), respectively (Figure 6). The PROTACs have been designed around a branched core (trifunctional natural amino acids), from where linkers connect two independent POI ligands and an E3 ligase binder. Among the CRBN-based PROTACs, compound DP-C-1 (**2**, Figure 6) displayed the best dual degradation profile, which was superior to that induced by the corresponding mono-PROTACs at the same concentration. Similarly, DP-V-4 (**3**, Figure 6) belonging to the VHL-based series, showed the best degradation effect, but a weaker anti-proliferative activity in tumor cells compared to the parent inhibitors. This was probably due to a poor PK profile resulting from the high MW. Engagement of both EGFR and PARP by **2** and **3** was confirmed, although no evidence on multiprotein complex formation was reported. Remarkably, this work is the first successful example of rationally designed *multitarget* PROTACs able to simultaneously promote degradation of two completely different POIs in tumor cells.

Along these lines, further rationally designed *multitarget* PROTACs (i.e., BET/HDAC degraders) have been reported,^60,61^ and we expect that others will show up soon.

An example of case (b) of Figure 5 encompasses the development of degraders based on a dual-targeting POI ligand (ABT-263, Figure 6). The anti-apoptotic BCL-2 family proteins (including BCL-xL, BCL-2, and MCL-1) are well-validated cancer targets, and dual inhibition is a promising therapeutic strategy.^62,63^ However, ABT-263, a potent dual BCL-2 and BCL-xL inhibitor, has not obtained regulatory approval due to its on-target thrombocytopenia. Thus, it was speculated that a PROTAC approach might avoid this side effect, since platelets express minimal levels of VHL, CRBN, and IAPs. To this end, DT2216 (**4**, Figure 6) was developed^64^ as the first PROTAC featuring a dual-targeting POI ligand. However, **4** did not show a dual degrader profile, while achieving only BCL-xL degradation. Thus, it had a limited effect on most solid tumors, unless it was combined with a selective BCL-2 inhibitor.^65^ From this clearly emerged the necessity of a simultaneous modulation of the two targets (BCL-xL and BCL-2) for a greater therapeutic impact. With the aim of developing a truly dual BCL-2/BCL-xL PROTAC, a different linker attachment point was explored. The most potent compound of the series, PZ703b (**5**, Figure 6), exhibited balanced potency in both MOLT-4 (BCL-xL-sensitive cell line) and RS4;11 cells (BCL-2-sensitive cell line). However, **5**-mediated BCL-2 degradation was not significant, although its inhibitory activity on BCL-2 was enhanced. A follow-up study^66^ allowed researchers to achieve the desired dual degradation profile. Guided by computational modeling of the entire multimeric ubiquitin ligase complex, **5** was effectively converted into a potent dual BCL-2/BCL-xL degrader, 753b (**6**, Figure 6), by modifying linker length and composition.^66^ A series of experiments confirmed that **6** degrades both BCL-xL and BCL-2 via the UPS. Remarkably, **6** showed a significantly improved anti-tumor efficacy in a Kasumi-1 acute myeloid leukemia (AML) cell line, which critically depends on both BCL-xL and BCL-2 for survival.

Collectively, these works address key aspects of *multitarget* PROTACs. Drug combinations of PROTACs and conventional protein inhibitors are more effective than PROTACs alone to perturb networks and modify the outcome of a complex disease. An even better modulation of pathological networks may be achieved by using single-molecule *multitarget* PROTACs, able to act at the same time and at the same concentration on the selected multiple targets (with respect to combinations, two single compounds, each one with an individual PK profile). However, *multitarget* PROTACs based on two different POI ligands (Figure 5A) are more challenging than classical PROTACs in terms of (i) synthesis and (ii) PK profiles, due to their inherently higher structural complexity. As for point (i), a toolbox for PROTAC modular synthesis^67^ and a “click chemistry platform” ^68^ have already proven effective for accessing libraries of PROTACs. In addition, branched functionalization sites with controlled orientation have been reported,^69^ clearly highlighting the ongoing interest of the scientific community to expand the field in this direction. As for the PK, due to the presence of a second POI ligand, *multitarget* PROTACs have an even higher MW than traditional PROTACs.^45^ Although PROTAC modes of cellular permeation/oral bioavailability are mostly unknown, the larger structure of *multitarget* PROTACs might pose further PK challenges.

With regard to PROTACs featuring dual-targeting POI ligands (Figure 5B), we should remark that besides the described examples, others have been reported.^70^ To note, all focus on PROTACs directed to proteins belonging to the same family. Clearly, it is more challenging to identify a chemical framework with a balanced activity against a set of multiple targets associated with a desired effect, which do not share binding site similarity.^71^

In addition to those of Figure 6, a particular case of dual-targeted activity is that shown by the so-called IRAKIMiDs.^72^ By exploiting the molecular glue activity of IMiDs, these single molecules have been designed to degrade both IRAK4 and IMiD substrates, including Ikaros and Aiolos. KT-413 (undisclosed structure), currently in a Phase I clinical trial, combines IRAK4 and IMiD degradation by simultaneously targeting both the MYD88-NFkB and IRF4-Type 1 interferon pathways. This should broaden the anti-tumor activity of this single agent.

All in all, the rational design of *multitarget* PROTACs remains a challenge, considering the unpredictable role of the ternary complex formation and the multifaceted cascade underlying targeted protein degradation. However, based on what we have learned from the MTDL field, we envision that the development of *multitarget* PROTACs might highlight that the *whole is greater than the sum of its parts* in terms of therapeutic outcome. *Multitarget* PROTACs might have better therapeutic windows, thanks to lower doses and the avoidance of drug–drug interactions, as well as reduced susceptibility to drug resistance. With this in mind, we hope that enriching PROTACs with polypharmacological modalities may not only open a new research direction but also foster clinical translation.

## 4 Combining PROTAC and Photopharmacology Modalities

“Photopharmacology” is a rapidly developing field that combines the classical pharmacological approach based on small-molecule drugs with the light control used in photochemistry.^48,73^ As such, photopharmacology aims at solving the problems of off-target activity and severe side effects by controlling drug activity with high spatiotemporal precision. Moreover, light can directly influence the action of bioactive molecules by changing their PK or pharmacodynamic (PD) profiles.^74^ To date, the field encompasses all approaches based on photoresponsive small molecules, including photocaged and photoswitchable ligands.^48,73^ Photocaged compounds are irreversibly photoresponsive small molecules decorated with a photocleavable protecting group (defined as “cage group”). The cage group masks the bioactive pharmacophore, thereby hampering the interaction with the desired target and making the molecule inactive. Then, photocleavage of the cage group upon irradiation allows the controlled activation of the bioactive compound, and thus its consequent pharmacological activity. Photoswitchable ligands are reversibly photoresponsive small molecules, capable of switching between isomeric forms. A light stimulus guides the reversible isomerization. The resulting conformational change enables on/off switching of the therapeutic action by affecting target recognition. From a medicinal chemistry point of view, both strategies have made tremendous progress in the past decade, with an extensive repertoire of photopharmacology small molecules directed to a wide array of biological targets.^48,75^

PROTACs could provide key advantages over classical inhibitors. However, their particular mechanism of therapeutic action might be associated with safety risks that could hamper “bench-to-bedside” advancement.^76^ When systemically administered, prolonged on-target protein degradation and associated POI loss of function might occur in any cell accessible to the degrader. For instance, inhibition of BET bromodomains is relatively tolerated, while a complete loss of BRD2 and BRD4 is lethal.^77^ To fine-tune PROTAC activity and avoid toxic events, an external stimulus might be highly beneficial. The controlled activation of PROTACs at a chosen time and location could indeed yield a potential better selectivity. Thus, several groups have been asking whether light could be used as a controllable stimulus, given its non-invasive action and high spatial and temporal precision.

**Figure 7:**
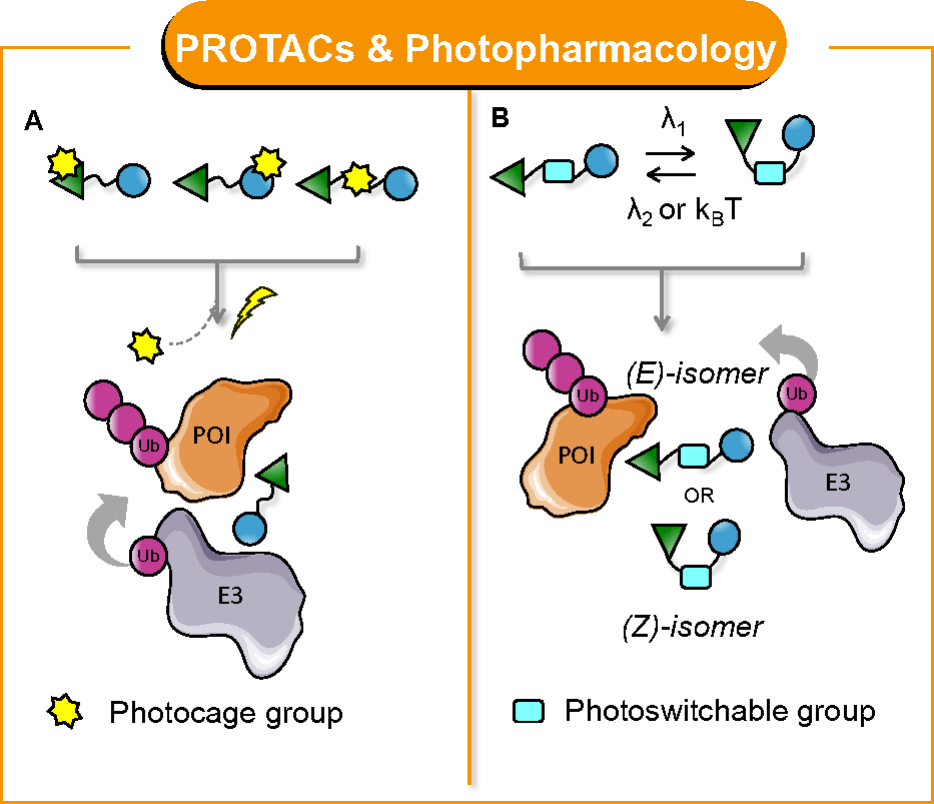
General structure and MoA of *light-controllable* PROTACs: (A) photocaged PROTACs and (B) photoswitchable PROTACs.

To answer such a question, several PROTACs have been converted into light-controllable precision tools. As discussed, the main outcome of such *multifunctional* PROTACs is the optical spatiotemporal control of protein degradation, which is generally related to the formation of a productive ternary complex upon irradiation. For clarity, we will group the developed light-controllable PROTACs into two categories, namely (i) photocaged PROTACs (Figure 7A) and (ii) photoswitchable PROTACs (Figure 7B).

### 4.1 Photocaged PROTACs

Typically, photocaged PROTACs utilize a photolabile protecting group that is irreversibly released upon light irradiation, leading to a tissue-selective activation of the degrader. The design and synthesis of a photocaged degrader are quite straightforward when starting from an already validated PROTAC. The incorporation of the cage group on the PROTAC structure can be performed by exploiting different chemical functionalization sites, or rather, by caging (i) the E3 ligase ligand to prevent crucial binding interactions with the corresponding protein, (ii) the warhead to impede POI recognition, or (iii) the linker to effect the protein–protein interaction between the POI and the E3 ligase and influence ternary complex formation (Figure 7A).

Among the three design options, the examples developed so far mainly involve approach (i). Thus, the cage group is primarily inserted on the imide nitrogen of the CRBN ligand or on the hydroxyl group of the VHL ligand, which should be restored—upon irradiation—to ensure E3 ligase molecular recognition.^78,79^ By caging the E3 ligase ligand, the degradation of the POI and potential off-target effects are abolished, while the uncaged warhead can still interact with its target and retain its full inhibitory activity (if any). On the other hand, by caging the warhead, either degradation or inhibition of the POI will be abrogated. In this case, the E3 ligase ligand, which interacts with CRBN, can still behave as a molecular glue, inducing potential E3-mediated off-target effects, like IKZF3 degradation.^80^

**Figure 8:**
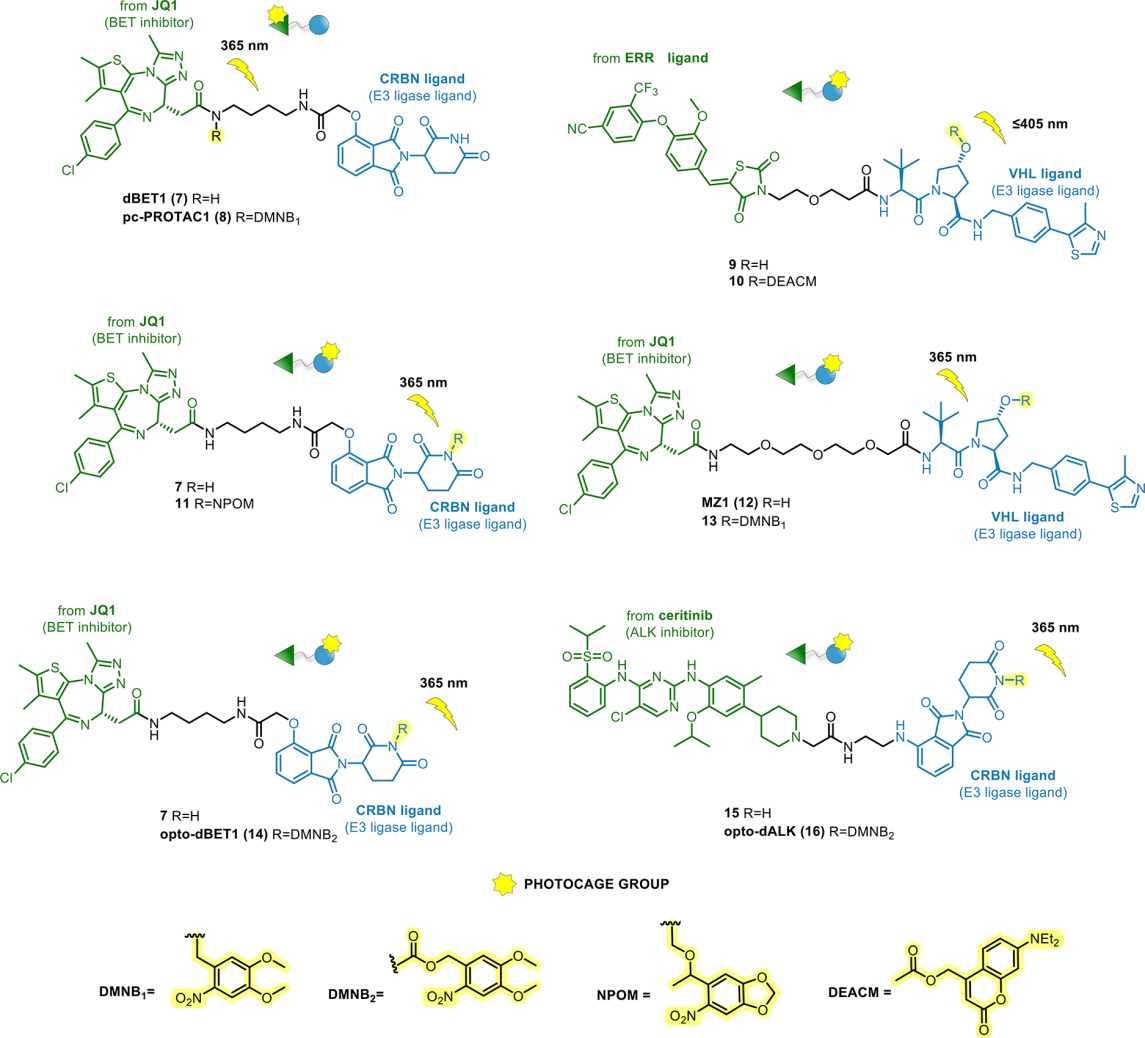
Design of photocaged PROTACs.

The first photocaged PROTACs have been conceived starting from a well-studied BRD2–4 degrader, dBET1 (**7**, Figure 8),^81^ which was functionalized with the appropriate photocage group. Xue et al. installed a 4,5-dimethoxy-2-nitrobenzyl (DMNB) cage group either on the CRBN ligand or on the amide connecting the bromodomain inhibitor JQ1 to the linker.^82^ This latter modification gave pc-PROTAC 1 (**8**, Figure 8), which was cleaved upon 365 nm irradiation to give the active **7**. **8** was demonstrated to bind BRD4 in the dark with more than 100-fold reduced affinity. Degradation of the POI was observed after a light irradiation of only 0.3 min, and the substrate was completely degraded after 3 min. This fascinating study culminated with an *in vivo* evaluation in zebrafish embryos, which confirmed the light-induced degrading activity of **8** at 50 and 100 μM.

In parallel, Naro et al. provided a general strategy to enable light-triggered protein degradation by any small-molecule warhead.^83^ To achieve that, they leveraged the strategic insertion of two different photocage groups on the E3 ligase ligands of parent CRBN-based (**7**)^81^ and VHL-based (**9**)^37^ PROTACs. In detail, **10** (Figure 8) was obtained from estrogen-related receptor α (ERRα)-targeted PROTAC **9** by inserting a diethylamino coumarin (DEACM) cage group on the hydroxyl of the VHL portion. Similarly, **11** (Figure 8) derived from **7** by the introduction of a 6-nitropiperonyloxymethyl (NPOM) moiety on the imide nitrogen of the CRBN ligand. The DEACM prodrug **10** showed efficient photolysis to release the active degrader **9** upon ≤405 nm light irradiation. The induced degradation was confirmed in MCF-7 breast cancer cells expressing ERRα transcripts, while showing no ERRα depletion in the dark. **11** was photoreleased from its cage group when irradiated with 365 nm light, restoring the BRD4-degrading ability of **7** in cells.

In line with these findings, Kounde et al.^84^ introduced the DMNB cage group to the VHL ligand of the BRD4-directed PROTAC MZ1 (**12**, Figure 8).^85^ As expected, the caged PROTAC **13** showed a dose-dependent degradation of BRD4 only upon irradiation at 365 nm, with a good stability profile in non-irradiated cells.

Finally, by inserting DMNB on CRBN ligand of parent PROTACs **7** and MS4048 (**15**),^86^ Liu’s group designed opto-dBET1 (**14**) and opto-dALK (**16**, Figure 8).^87^ Biochemical and biological evaluations showed the light-inducible (365 nm) degradation of both target proteins (BRDs and ALK fusion protein) in a timely and dose-dependent fashion.

Collectively, we would like to emphasize how the photocaging strategy can be a universal technology for developing *light-controllable* PROTACs. In particular, the caging of E3 ligase ligands seems, to date, the most feasible approach which is worthy of future applications.

### 4.2 Photoswitchable PROTACs

The idea of combining light and P-mPD is also embodied in photoswitchable PROTACs. Photoswitchable degraders undergo a geometrical conformational modification upon light irradiation, which subsequently enables the reversible on/off switching of protein degradation. Commonly, by incorporating a photoswitch unit into a biologically active compound, light can be used to “switch” the molecule between two states with different binding affinities to the target (i.e., “on” and “off”).

To date, the prototypical azobenzene photoswitch has been largely used for the development of photoswitchable PROTACs, given its chemical stability, predictable geometrical changes (*Z*/*E* isomers), and facile modulation of properties.^88^ Moreover, incorporation of an azobenzene moiety into a PROTAC structure does not lead to a significant increase of in the MW of the final molecule. Clearly, the switch from *Z* to *E* isomer affects binary and ternary complex formation. Overall, the *Z* isomer is generated upon irradiation of its *E* counterpart using a selected wavelength (λ_1_), while the thermodynamically more stable *E* isomer is obtained from *Z* with another specific wavelength (λ_2_) or by thermal relaxation (*k*_B_*T*). The resultant light-guided and reversible conformational change would then enable a distinct degradation profile for each PROTAC isomer (Figure 7B).

**Figure 9:**
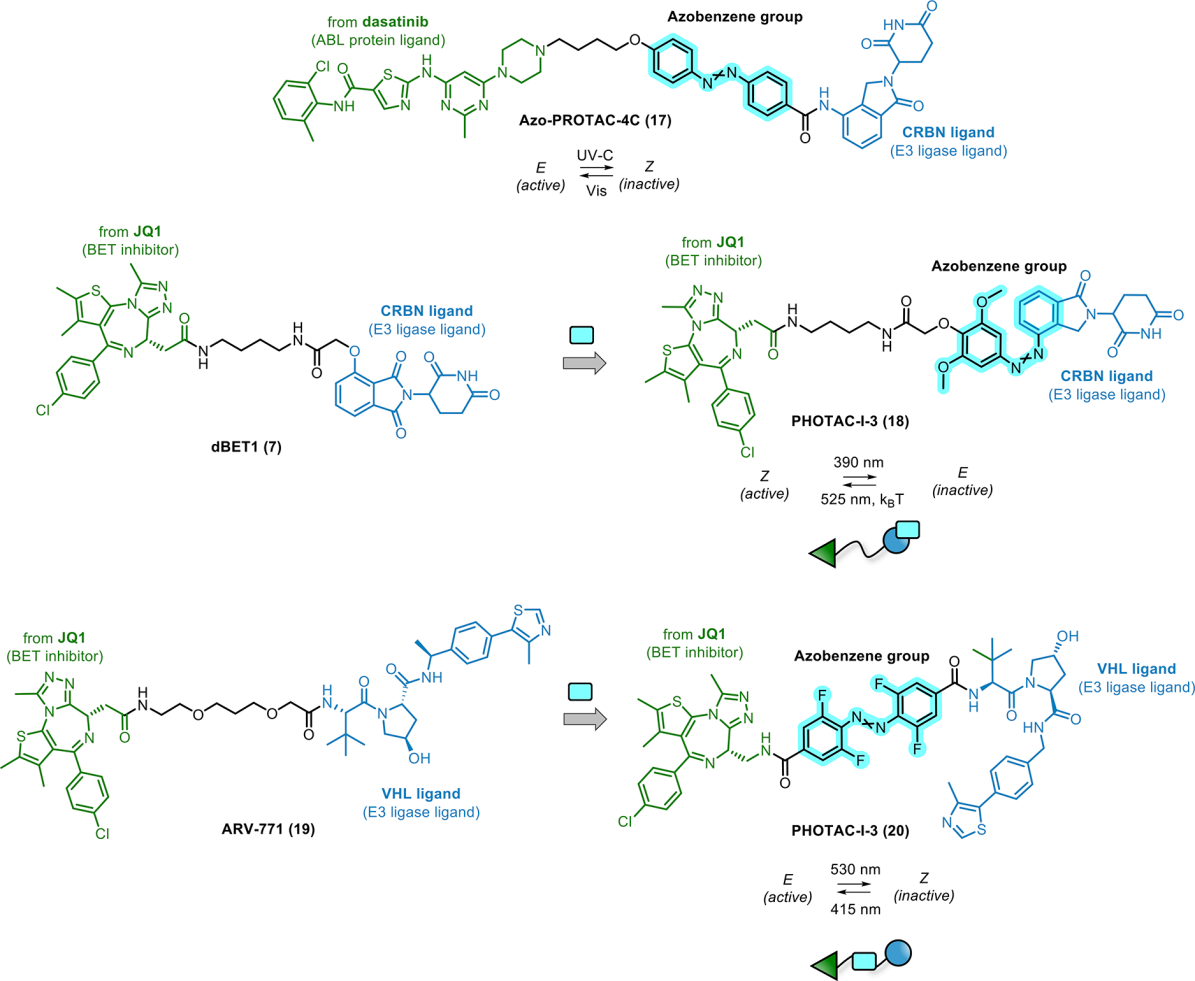
Design of photoswitchable PROTACs.

Jin, Lu, et al. rationally designed a small series of photoswitchable PROTACs by attaching the azobenzene unit at the phenyl ring of lenalidomide.^89^ Starting from the X-ray crystal structure of CRBN in complex with its binder, they assumed that the two azobenzene configurations would affect its binding and the subsequent degradation profile of the lenalidomide-based PROTAC. By connecting the azobenzene-derived lenalidomide to a BCR-ABL inhibitor dasatinib, they came up with AZO-PROTAC-4C (**17**, Figure 9) as a reversible and light-inducible PROTAC. They demonstrated that the *E* and *Z* isomers of **17** had significantly different protein degradation profiles in cells. Specifically, by using a BCR-ABL-positive K562 cell line, the *E* isomer degraded BCR-ABL fusion protein and ABL, whereas no degradation was detected using the *Z* isomer. However, the geometrical switch to the active **17** and the related degradation outcome were controlled only by UV-C light irradiation. As it is well known, UV-C light (200–280 nm) is characterized by poor penetrability and harmful effects on cells, which precludes or greatly hampers future development and use.

By following a similar chemical functionalization strategy, Reynders et al. inserted the azobenzene unit as part of lenalidomide of **7**, developing a series of photoswitchable PROTACs targeting several POIs, including BET (BRD2–4) proteins.^90^ To note, the BRD2–4-directed PROTAC, PHOTAC-I-3 (**18**, Figure 9), emerged as one of the most potent light-controllable degraders. In detail, upon irradiation with 390 nm light pulses, **18** rapidly isomerized to the active *Z* form, and consequent BET protein degradation was detected in AML cell line RS4;11. However, only a slight recovery of protein levels was observed after 525 nm irradiation or compound thermal relaxation in the dark (*Z*-to-*E* isomerization).

By following a rational design, Crews’s lab achieved a fine spatiotemporal control of BRD4 degradation.^91^ In a previous report, the authors had demonstrated that the difference in linker length between active (11 Å) ARV-771 (**19**, Figure 9)^92^ and inactive (8 Å) PROTACs was critical for inducing BRD4 degradation. This was confirmed in the corresponding photoPROTAC-1 (**20**, Figure 9), obtained by inserting an *ortho*-tetrafluoro-azobenzene in the linear PEG linker of **19**. Accordingly, the corresponding *E* and *Z* isomers showed a dramatic difference in promoting BRD4 degradation. The *Z***-20** was inactive, probably because of the unsuitable distance between BRD4 and VHL ligands to engage both proteins in a ternary complex. By contrast, the *E-***20** isomer turned out to be an active degrader, facilitating the ubiquitination of the POI. Moreover, a previous report showed that *ortho*-tetrafluoro-azobenzene motif generated thermally bistable photoswitches,^88^ meaning that the optimized light-controllable PROTACs were stable in both conformations. In this case, irradiation with 530 nm light gave the inactive *Z***-20**, while irradiation with 415 nm generated the active *E*-**20**. As a result, the possibility to avoid laborious continuous irradiation represents valuable progress in such photoactivable technology in terms of potential translation.

From what was discussed above, both photocaged and photoswitchable PROTACs are valid examples of how photopharmacology enables on-demand protein degradation. Irreversibly photocaged PROTACs activate the P-mPD without the possibility to reverse the degradation event. Caging the E3 ligase ligands, as in the reported case studies, could be exploited to easily transform a PROTAC into a light-controllable one. However, it should be noted that conjugation of a photocage group causes an increase of the MW of the prodrug, which might result in unfavorable drug-like properties. On the contrary, photoswitchable motifs are not released after photoactivation. Moreover, the incorporation of a photoswitch into PROTACs (either on E3 ligase ligands, POI ligand, or linker) requires a more iterative structural modification. Even in this case, insertion of the photoswitch moiety into the E3 ligase ligand may provide a more modular and general approach. However, potential PROTAC linkers, such as those incorporating stilbenes, 1,2-diphenyl ethanes, 1,2-diphenyl hydrazines, *N*-benzyl anilines, benzyl-phenyl ethers, benzyl-phenyl thioethers, diaryl esters, diaryl amides, and heterocyclic derivatives thereof,^93^ might be suitable targets for azologization.

In conclusion, although *light-controllable* PROTACs offer promise for spatiotemporal control of protein degradation and side effect minimization, they still face clear limitations. Particularly, their clinical translation may be hampered by light-induced cellular damage and poor tissue penetration of the incident light.

## 5 Combining PROTAC and Drug Conjugate Modalities

In recent decades, targeted delivery of therapeutic agents to diseased cells and tissues, and not to healthy ones, has been made possible by nanotechnology approaches^94^ or by drug conjugates featuring a targeting moiety.^95^ Antibody–drug conjugates (ADCs) are generally comprised of an antibody (Ab) conjugated to a cytotoxic payload via a chemical linker.^96^ Three ADC therapeutics (trastuzumab emtansine, brentuximab vedotin, and inotuzumab ozogamicin) are already on the market, and several others are being investigated in clinical trials.^97^ More recently, small molecule–drug conjugates (SMDCs) have provided an opportunity for targeted delivery. Typically, SMDCs feature three parts, i.e., a targeting ligand, a cleavable linker, and a therapeutically active small molecule. Targeted drug conjugates act by recognizing target cells through overexpressed receptors and, once internalized, selectively releasing the therapeutic agent. In most cases, release occurs following linker cleavage by intracellular thiols^98^ or by using self-immolative linkers responsive to specific stimuli, such as pH, redox system, and light.^94,99^

**Figure 10:**
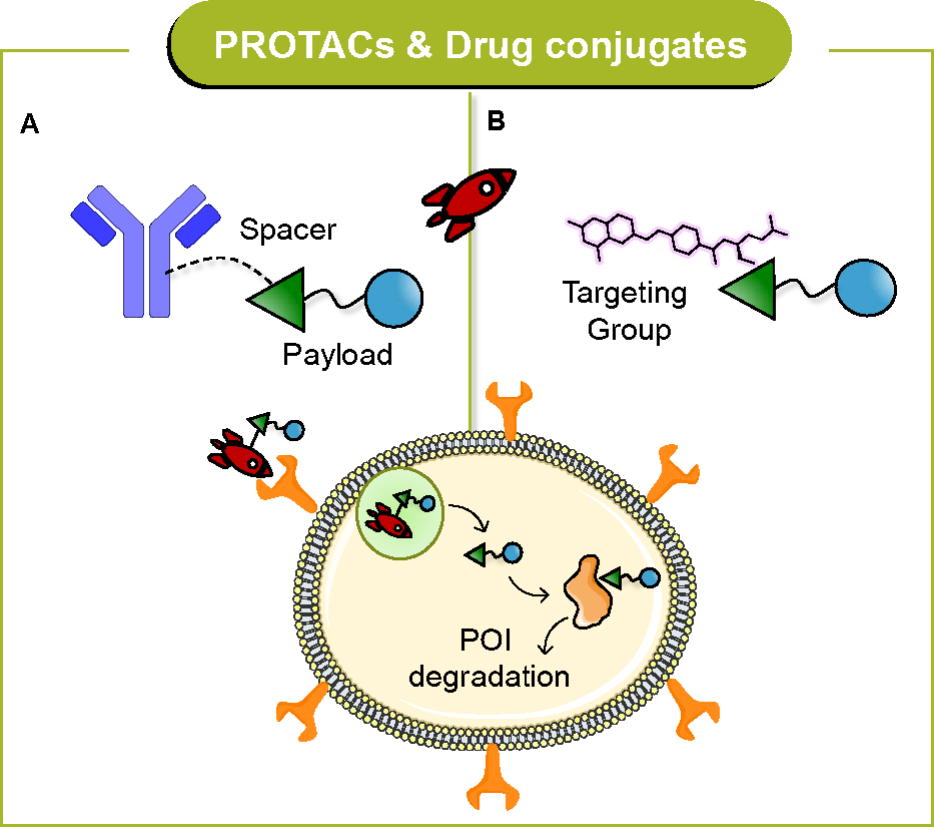
General structure and MoA of *PROTAC conjugates* for targeted delivery: (A) antibody-PROTAC conjugates and (B) small molecule-PROTAC conjugates.

As mentioned earlier, PROTACs effect highly efficient protein degradation and represent a unique therapeutic strategy. However, when addressing target proteins expressed in most of tissues, PROTACs may lack selectivity. Clearly, tissue- or organ-specific degradation can be achieved by exploiting differential biology and expression levels of E3 ligases.^35^ Another possibility is the development of a site- or tissue-specific delivery system that can reduce potential toxicity and increase the therapeutic window and eventually the translatability into the clinical setting. Thus, PROTAC conjugates have attracted considerable attention for targeted delivery (Figure 10).

In the following sections, (i) antibody- (Figure 10A) and (ii) small molecule-PROTAC conjugates (Figure 10B) will be discussed from the viewpoint of a double modality.

### 5.1 Antibody–PROTAC Conjugates

ADCs are targeted drug conjugates formed by three main parts: the Ab as the targeting moiety, the therapeutic agent (payload), and the spacer connecting the Ab to the payload. In this way, the high toxicity of the cytotoxic small-molecule payload is combined with the high selectivity of the Ab.^100^

Once administered, ADCs bind to the target cell through specific antigen recognition and release the active molecule inside the cell, producing the desired therapeutic effect. Although groundbreaking, there are some important factors that need to be considered when designing an ADC.^100^ First is the identification of the target antigen. To limit off-target effects and therefore toxicity, the target antigen should be overexpressed in diseased cells, with little or no expression in normal tissues. Second, the choice of the Ab should be dictated by the high specificity for the target antigen to avoid cross-reactions and off-target toxicity.^101^ Spacer optimization is another element to take into account. Spacers in ADC must be stable during systemic circulation but then able to be cleaved and release the payload once the ADC is internalized into the target cell. Generally, especially in case of cancer applications, the payload needs to fulfill the following requirements: (i) to be highly active (IC_50_ value in the low nanomolar or picomolar range), (ii) to act through a well-defined MoA, and (iii) to have a suitable attachment point for spacer insertion.^102^ Another critical aspect is the drug–antibody ratio (DAR), i.e., the number of therapeutic molecules loaded onto the Ab. Clearly, the potency of ADC increases with higher levels of drug loading. This can, in turn, affect the stability, PK, and toxicity profile of ADCs. In addition, many payloads used in ADCs are hydrophobic in nature and thus are prone to Ab aggregation, which must be avoided to ensure a suitable shelf life and to limit fast clearance and immunogenicity.^100^ Although most of the developed ADCs focus on cancer treatment, the idea is also being explored outside the oncology field.^103^

In this scenario, there is a growing interest in exploring conjugation of PROTACs to Abs, by implementing strategies previously developed for other payloads.

**Figure 11:**
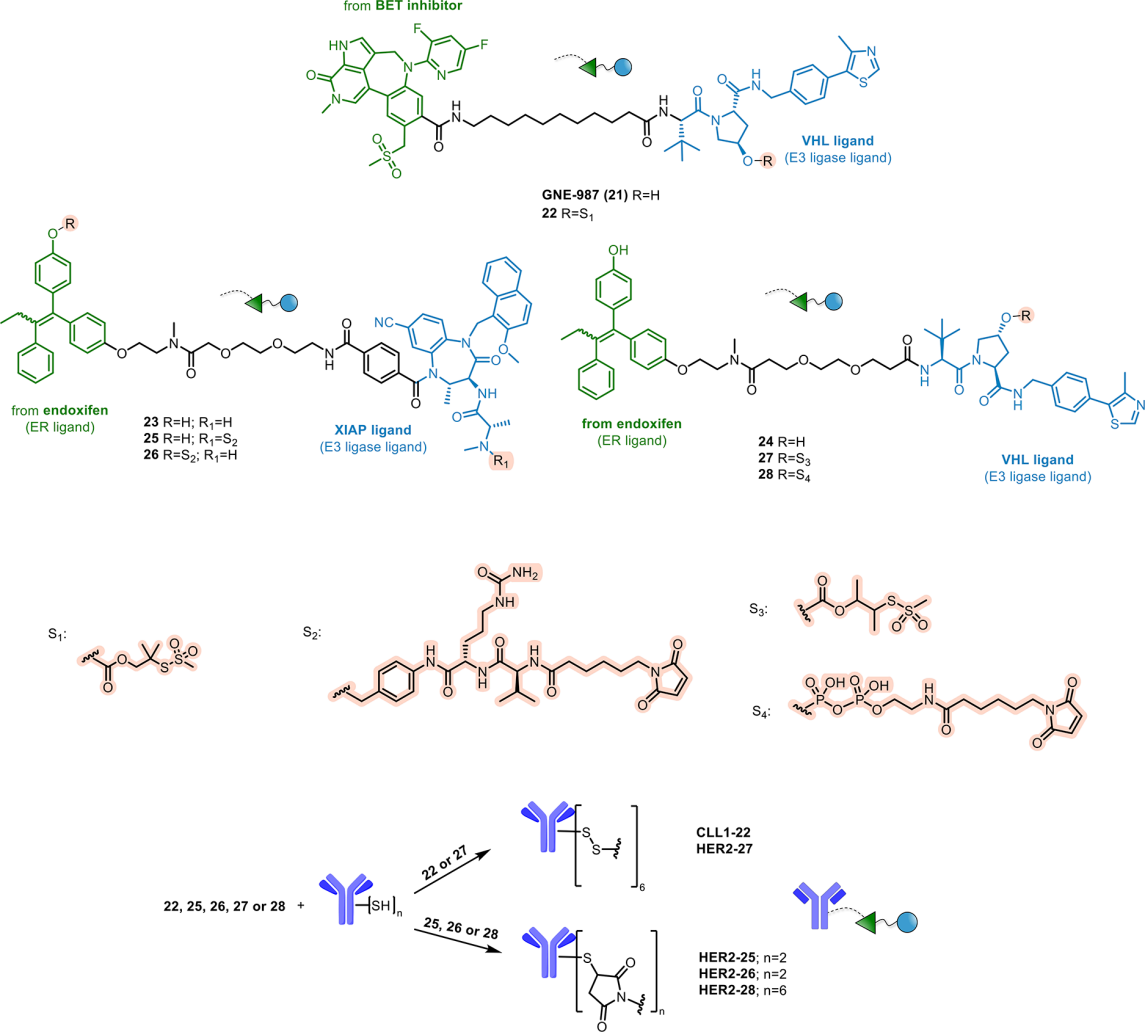
Design of antibody–PROTAC conjugates.

Dracovich et al. developed the first reported Ab-PROTAC conjugate by attaching a BET degrader to an anti-C-type lectin-like molecule-1 (CLL1) antibody.^104^ CLL-1 is an ideal target for an Ab-based therapy for AML, due to its high expression in leukemic cells, while being absent in normal hematopoietic stem cells. The authors synthesized a new potent BRD4 degrader, GNE-987 (**21**, Figure 11), by incorporating the VHL-binding moiety along with the structure of a potent BET inhibitor. **21** turned out to be a potent degrader, which exhibited picomolar potency in a cell model of AML (EOL-1 AML). However, it demonstrated unfavorable *in vitro* PK properties, confirmed by a poor *in vivo* PK profile after intravenous or oral administration in mice. To overcome these issues, **21** was modified by inserting, on the VHL moiety, the S_1_ disulfide-containing cleavable spacer to provide **22** (Figure 11), which, in turn, was conjugated to a CLL1-targeting Ab, leading to the Ab-PROTAC conjugate **CLL1-22**. Following intravenous administration in a xenograft mouse model of AML overexpressing CLL1, **CLL1-22** showed high dose-dependent potency, stronger than that of **21**. Moreover, the intratumor levels of **21** well correlated with the **CLL1-22** administered dose. The minimal activity of the epimer of **21**, not able to bind to VHL E3 ligase, confirmed that the *in vivo* efficacy was related to BET degradation. **CLL1-22** also exhibited favorable *in vivo* stability and improved PK, validating the design rationale. The systematic development of **21** and related Ab-PROTAC conjugates, including the optimization of the BRD4-binding fragment and the use of different Ab spacers, is described in two subsequent papers to which the reader is referred for further details.^105,106^

In another study, the same research group reported Ab-PROTAC conjugates obtained by attaching two different estrogen receptor α (ERα) degraders (**23** and **24**, Figure 11) with anti-human epidermal growth factor receptor 2 (HER2) Ab, through three spacer modalities (S_2_, S_3_, and S_4_, Figure 11).^107^ In a first attempt, they derivatized PROTAC **23** on the E3 ligase ligand with a valine-citrulline-*para*-amino-benzyloxy spacer (S_2_, Figure 11) obtaining **25** (Figure 11) and the respective DAR2 Ab-PROTAC conjugate **HER2-25** (Figure 11). However, although **HER2-25** was successfully synthesized, it presented a high level of self-aggregation. To overcome this issue, they connected the S_2_ to the phenol group of endoxifen in **23**, affording the spacer-PROTAC **26** and the corresponding DAR2 conjugate **HER2-26**. The latter was characterized by reduced aggregation potential and efficient antigen-dependent delivery in a breast cancer cell line overexpressing HER2. Despite these encouraging data, **HER2-26** proved to be unstable upon *in vivo* administration. Thus, starting from **24**, spacer-PROTACs **27** and **28** (Figure 11) were synthesized by inserting a disulfide spacer (S_3_) or a pyrophosphate diester (S_4_) and then derivatized to the corresponding Ab-PROTACs **HER2-27** and **HER2-28** (Figure 11). These Ab-PROTAC conjugates showed moderate stability in mice, retaining 80% of the original DAR value after 72 h.

**Figure 12:**
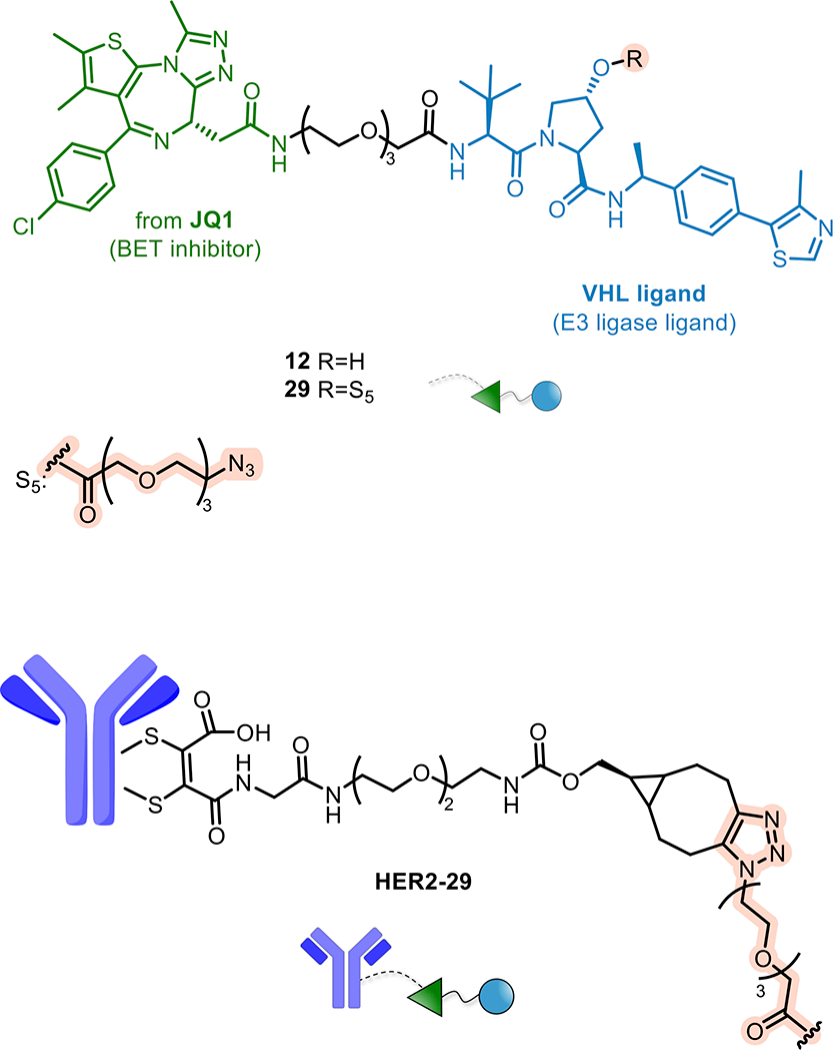
Design of antibody-PROTAC conjugate **HER2-29**.

Another research group developed an Ab-PROTAC conjugate (**HER2-29**, Figure 12) by combining the S_5_ azido-PEG spacer with the BRD4 degrader **12**. The resulting spacer-PROTAC (**29**) was easily conjugated to dibromomaleimide-strained alkyne-functionalized anti-HER2 Ab through a copper-free click chemistry reaction.^108^ DAR4 **HER2-29** showed excellent stability in PBS and selective degradation of BRD4 only in HER2+ cells, leaving BRD4 levels intact in HER2– cell line. Experiments performed in the presence of proteasome inhibitors confirmed that the degradation was proteasome-dependent, providing the cellular proof-of-concept for antigen-specific delivery and targeted protein degradation.

**Figure 13:**
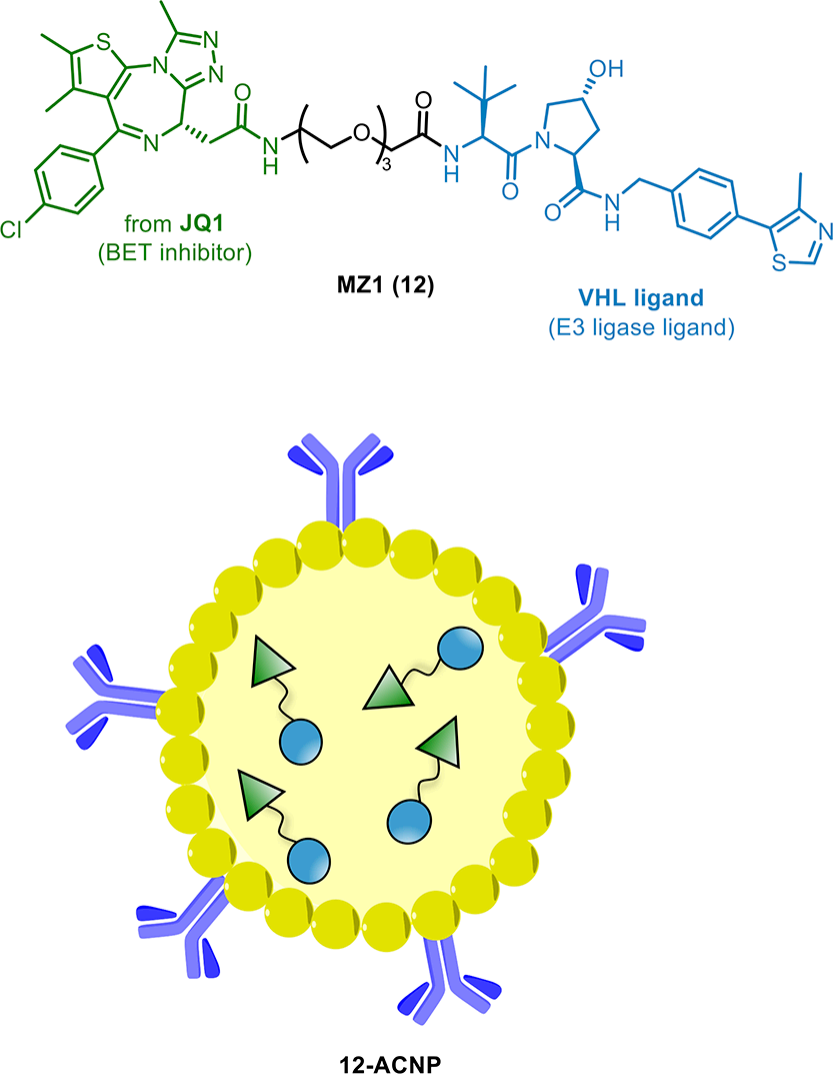
Design of **12** and **12-ACNP**.

As an evolution of the Ab-PROTAC conjugate concept for the treatment of breast cancer, the well-studied **12** was encapsulated into Ab conjugate nanoparticles (ACNPs).^109^ The authors selected poly-lactic acid and polyethylenimine as building blocks for the preparation of the nanoparticles, which were loaded with **12** and conjugated with the anti-HER2 Ab trastuzumab via a covalent bond (Figure 13). The resulting ACNPs (**12-ACNP**, Figure 13) were characterized by an improved anti-tumoral efficacy in HER2+ overexpressing breast cancer cell lines when compared to **12** and exhibited high stability and controlled release over time. Conjugation did not modify the MoA of **12** and did not lead to additional toxicity and side effects. Importantly, **12-ACNP** displayed a strong cytotoxic effect in trastuzumab- and **12-**resistant HCC1954 cell lines, overcoming the resistance developed to this degrader.

From the reported examples, it emerges that the selective antigen-dependent delivery of Ab-PROTAC conjugates could enhance the selectivity of the loaded PROTACs and reduce their side effects. Despite these encouraging remarks, there are some issues that cannot be overlooked. The main drawbacks of Ab-PROTAC conjugates are their relatively high tendency to aggregate, immunogenicity, and instability during systemic administration, which clearly limit their effective application in the clinical setting.^100^

### 5.2 Small Molecule–PROTAC Conjugates

Given the current limitations of Ab-PROTAC conjugates, targeted delivery by SMDCs seems an attractive alternative. Compared to Abs, small molecules usually have a better *in vivo* PK/PD profile and no immunogenic issues. In addition, they are easily obtainable by chemical synthesis, with a relatively cheap cost, and have a superior stability/shelf life.^110^ On the other hand, owing to their reduced size compared to Abs, small molecules are likely to possess lower affinity and specificity as targeting moieties, although certain exceptions may exist.^49^

Most small-molecule targeting moieties bind to specific receptors overexpressed in the diseased tissue or are sensitive to specific tissue conditions (e.g., hypoxia). For the first application, vitamin B12 and transcobalamin receptor,^111^ transferrin and transferrin receptor,^112^ and folate and folate receptor^113^ have been mainly used as targeting moieties and coupled receptors. Exploiting a small-molecule-based targeting strategy may overcome the somehow unselective tissue profile of the starting PROTAC while maintaining the advantages of a small molecule. In the following subsection, selected examples of small molecule–PROTAC conjugates (Figure 10B) will be used to substantiate this concept.

**Figure 14:**
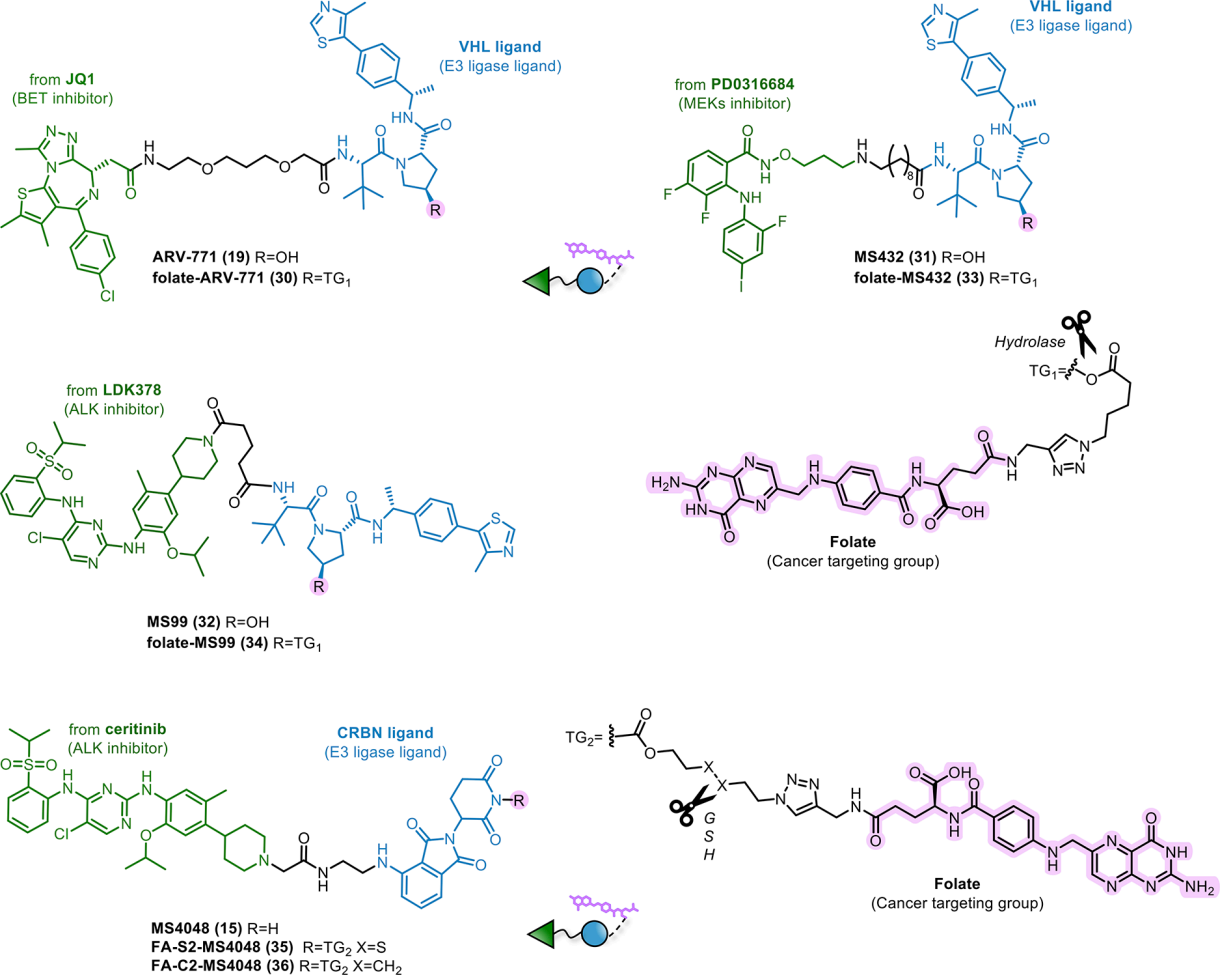
Design of small molecule–PROTAC conjugates.

To reach a better selectivity profile on tumor cells, the insertion of a folate group on a PROTAC structure has been recently investigated.^114,115^ Folate binds to its folate α receptor (FOLR1) that is highly expressed in various cancer cells and not in normal cells.^113^ Thanks to this specificity, FOLR1-targeted drugs are currently in Phase II/III clinical trials,^113,116^ and a FOLR1-targeted imaging diagnostic agent has been recently approved.^117^ By inserting the folate moiety on the E3 ligase ligand, folate-caged PROTACs were developed to initially mask the E3 ligase motif. As a prodrug strategy, only once internalized, the folate-caged PROTAC is activated after being released by endogenous hydrolase cleavage. In detail, based on the well-studied ARV-771 (**19**, Figure 14), Jin and colleagues^114^ developed the corresponding folate-caged PROTAC (**30**, Figure 14) by functionalizing the hydroxyl group of VHL via an ester bond. As discussed in section 4.1, the hydroxyl group of the VHL ligand is crucial for the recruitment of E3 ubiquitin ligase.^79^ The targeted delivery by such folate conjugation can provide a highly specific degradation due to both selectivity for tumor cells and activity triggered only after internalization and cleavage. This was experimentally confirmed by the design of an uncleavable (amide bond) folate-caged PROTAC. To evaluate tissue selectivity, **30** was tested in three cancer cell lines overexpressing FOLR1 (HeLa, OVCAR-8, and BRCA cells) and three non-cancerous cell lines (HFF-1, HK2, and 3T3). The results demonstrated that **30**, as well as **19**, efficiently degraded BRD4 in all three cancer cell lines. Remarkably, folate-caged **30** showed a less efficient degradation than **19** in normal cell lines not overexpressing FOLR1. To prove whether folate conjugation was the only factor mediating the PROTAC targeted delivery, competition studies with free folic acid were performed in HeLa cells. Folic acid could indeed antagonize the ability of **30** in degrading BRD4, but not that of **19**. In the same study,^114^ starting from mitogen-activated protein kinase (MEK1/2) PROTAC MS432 (**31**) and ALK PROTAC MS99 (**32**, Figure 14), two further folate-caged PROTACs (**33** and **34**, Figure 14) were developed. Similarly, it was shown that MEK1/2 and ALK were selectively degraded in cancer cells, in a folate receptor-dependent fashion.

Based on MS4048 (**15**, Figure 14),^86^ the same group developed a folate-caged PROTAC FA-S2-MS4048 (**35**, Figure 14).^115^ The folate group was inserted on the glutarimide core of pomalidomide via a self-immolative linker, initially hindering the interaction with the E3 ligase. The presence of a disulfide bond, which is cleaved by the intracellular glutathione (GSH), triggers the release of the active, uncaged **15**. Compared to the other self-immolative linkers, disulfides have demonstrated high therapeutic performance in terms of biocompatibility, stability, and selective cleavage by the high level of GSH in the cytoplasm.^98^ For this reason, **35** remains stable in the oxidized state until cell penetration and is subsequently cleaved by GSH thiol after internalization. The key role of GSH in releasing the active PROTAC was experimentally confirmed (i) by pretreating cancer cells with *S*-acetyl-l-glutathione (*S*-Ac-GSH) to supplement the intracellular GSH level or (ii) by exchanging the disulfide moiety with a methylene one. In the latter case, FA-C2-MS4048 (**36**, Figure 14), which remains caged after entering the cells, showed no activity. Conversely, **35** was shown to degrade ALK fusion proteins effectively and selectively in FOLR1-expressing cells, and in a FOLR1-, CRBN-, and proteasome-dependent manner. However, the authors stressed how the conjugation, causing a MW increase of ca. 1000 Da, might compromise the PROTACs’ PK properties.^114^

Another reported targeted delivery strategy relies on cage moieties responsive to specific cellular conditions. Hypoxia is a hallmark of most solid tumors and directly correlates with levels of nitroreductases (NTRs). On the contrary, NTRs are only expressed to a low level in normal tissues. Thus, prodrugs carrying nitroaromatic trigger units have been widely used.^118^ Nitroaromatic groups can be selectively reduced by NTRs to hydroxylamine and release the active drug after intramolecular rearrangement. The anti-tumor drug evofosfamide (TH-302) is a bromo-isophosphoramide mustard prodrug bearing a nitroaromatic group, which has entered Phase III clinical trial with promising results.^119^

**Figure 15:**
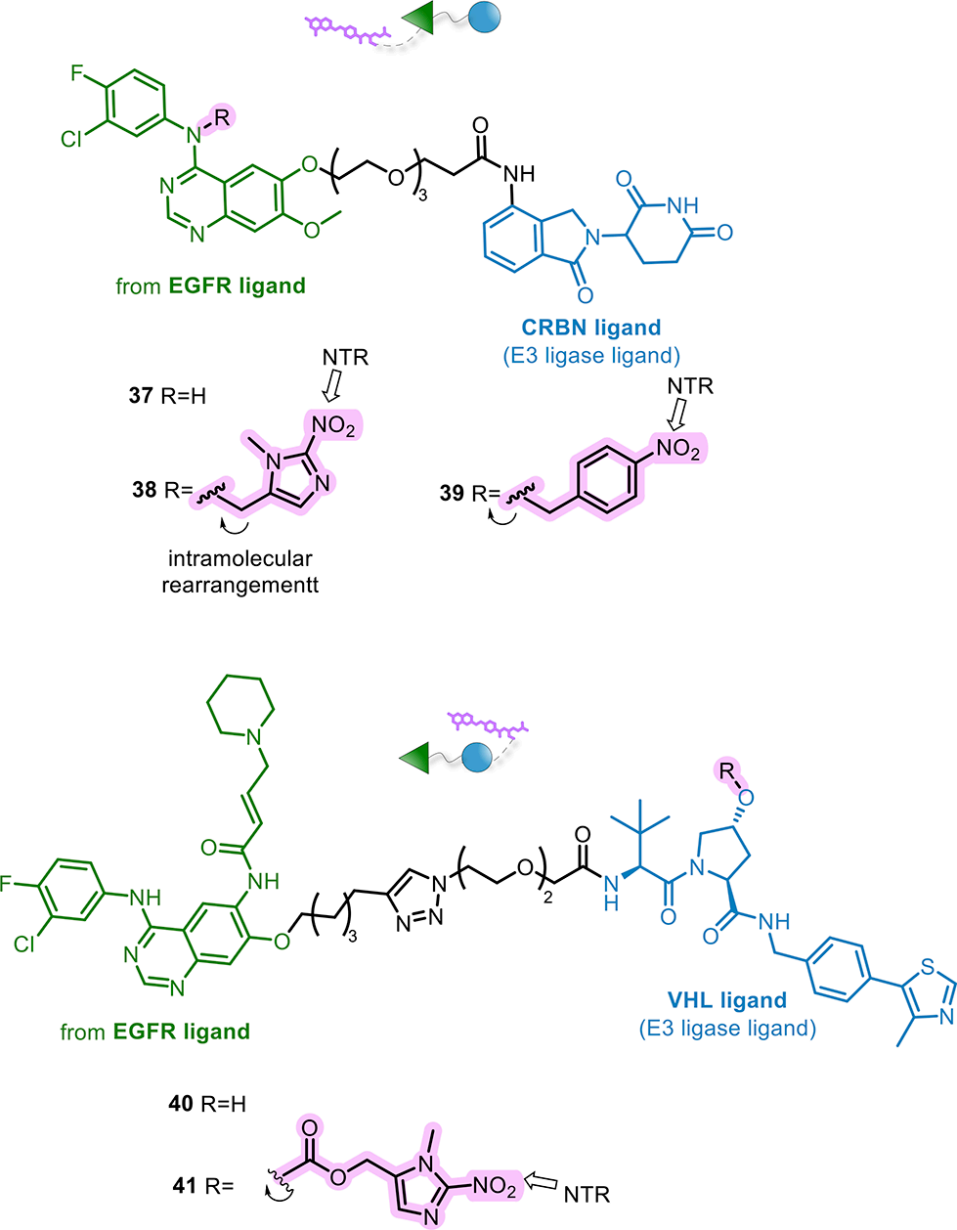
Design of caged PROTACs responsive to specific cellular conditions.

In 2021, Tian and co-workers^120^ applied a hypoxia-activated prodrug strategy by inserting nitroaromatic groups into PROTAC structures. Starting from the EGFR-directed degrader **37** (Figure 15), they developed PROTAC prodrugs **38** and **39** by introducing nitroaromatic moieties into the POI ligand (Figure 15). As prodrugs, **38** and **39** did not recognize EGFR due to the steric hindrance of (1-methyl-2-nitro-1*H*-imidazol-5-yl)methyl and 4-nitrobenzyl groups. In fact, **38** and **39** showed a POI degradation activity that was significantly higher in hypoxic cells than in normal ones. The active PROTAC **37** was selectively released after NTR bioreduction and intramolecular rearrangement, to exert its degrading effect.

A similar hypoxia-activated prodrug strategy was also investigated by Zhu et al. in a very recent work.^121^ In this case, the authors incorporated a (1-methyl-2-nitro-1*H*-imidazol-5-yl)methyl group on the hydroxyl of VHL ligand. Starting from the EGFR-directed PROTAC **40**, they synthesized the PROTAC prodrug **41** (Figure 15), which as a result was unable to degrade EGFR under normoxic conditions. Conversely, it was effective in NTR overexpressing tumor tissues. Notably, **41** induced EGFR degradation and exerted anti-tumor effects *in vivo*. Once more, given the wide use of the VHL ligand in PROTAC development, the insertion of the cage group at this level could find broad applicability.

As a general consideration on this combined modality, it should be noted that not all the reported examples have been validated *in vivo*, while their proof-of-concept is usually restricted to a cellular setting. Therefore, further translational studies are needed to confirm these small molecule–PROTAC conjugates as therapeutic tools able to improve PROTAC specificity, selectivity, and potency, while decreasing toxicity.

## 6 Combining PROTAC and Macrocycle Modalities

“Macrocycles” is an umbrella term for a diverse group of molecules, such us cyclic small molecules or cyclopeptides.^1^ As novel chemotypes with a MW of 500–2000 Da, macrocycles cover a chemical space beyond traditional medicinal chemistry strategies and fill an important gap between small molecules and larger biologics.^1^ Macrocyclic drugs have drawn significant attention due to their high selectivity, capability to target protein surfaces traditionally considered “undruggable”, and improved synthetic feasibility.^50,51^ In addition, in the case of biologically active peptides, macrocyclization may improve metabolic stability, cell permeability, and oral bioavailability. For these reasons, in recent decades, macrocyclic molecules have emerged as an attractive new therapeutic modality.^27,122^ From a medicinal chemistry viewpoint, macrocyclic structures can derive from natural cyclopeptides or as a result of a macrocyclization strategy. In the first case, structurally diverse and complex naturally derived macrocycles have demonstrated an impressive record of efficacy as pharmaceutical agents and are playing an increasingly important role in the treatment of a range of serious diseases.^123^ In the second case, macrocyclization is a design strategy for locking a known binder into its bioactive conformation and improving its PD profile.^124^ Already 19 macrocyclic drugs, including three radiopharmaceuticals, have been approved by the FDA for the treatment of infectious and metabolic diseases, cancer, immunosuppression, etc.^125^

**Figure 16:**
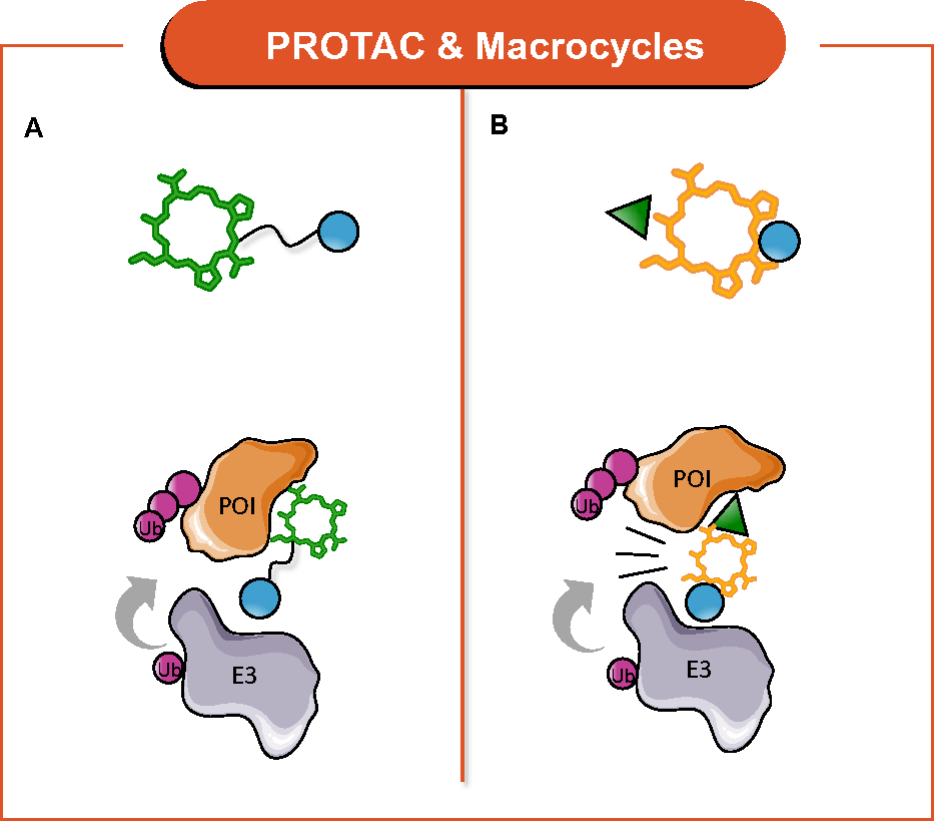
General structure and MoA of *macrocycle-based* PROTACs: (A) macrocyclic molecules as POI ligands and (B) macrocyclization of PROTACs.

Here, we review recent examples on how (i) macrocyclic molecules (Figure 16A) and (ii) macrocyclization strategies (Figure 16B) have been fused with the PROTAC concept to provide the so-called *macrocycle-based* PROTACs.

**Figure 17:**
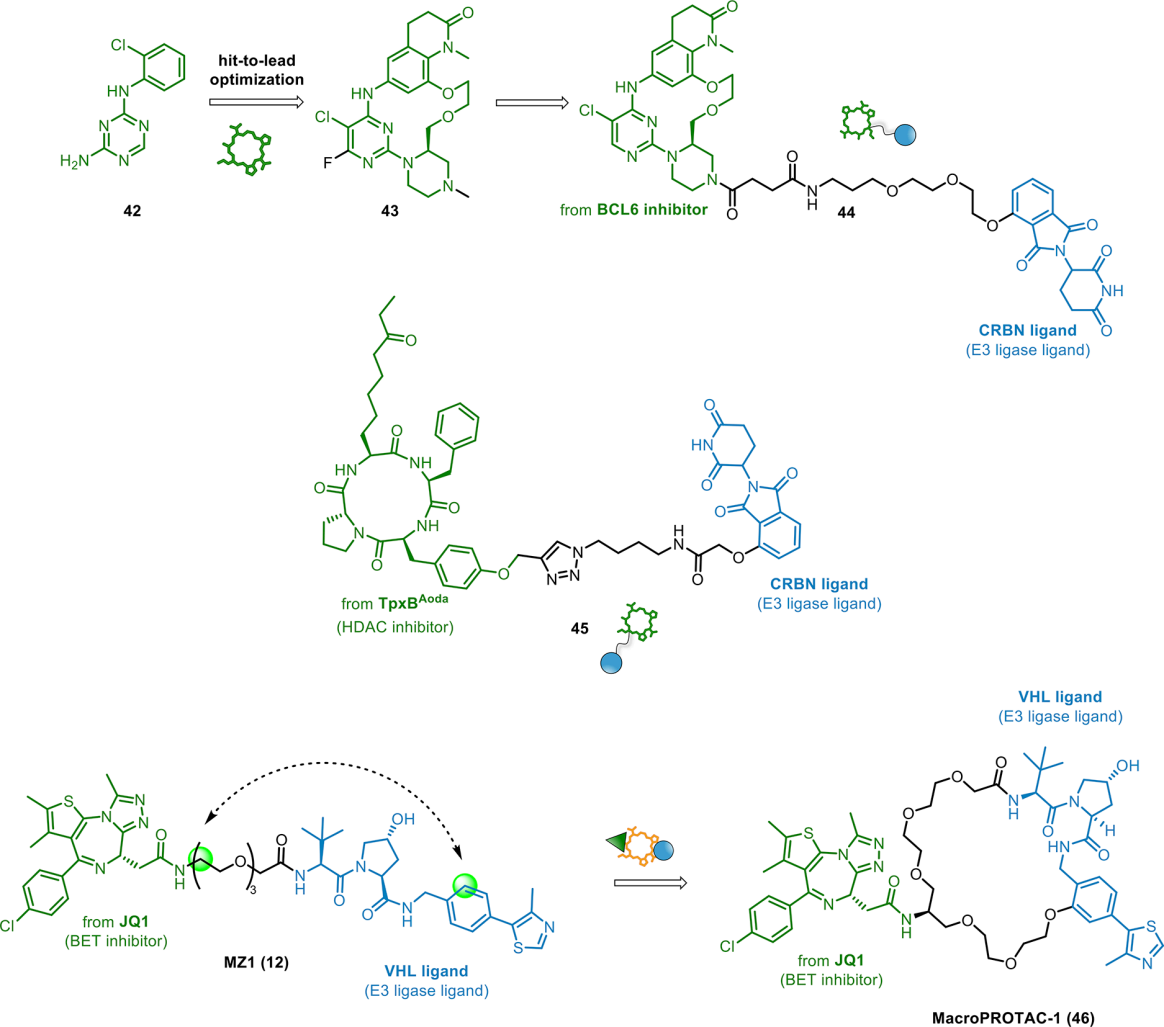
Design of macrocyclic-based PROTACs.

Macrocycle-based therapeutic modality was first combined with PROTAC technology by McCoull’s group, which developed macrocyclic molecules as B-cell lymphoma 6 (BCL6) protein inhibitors.^126^ A hit-to-lead optimization campaign was pursued on fragment-like hit **42**, giving rise to macrocycle **43**, which demonstrated high activity, good cellular potency, in-cell target engagement, and excellent selectivity (Figure 17). The design strategy of BCL6 binder **43** was guided by NMR-based conformational analysis. Then, macrocycle-based PROTAC **44** (Figure 17) was obtained through the conjugation of **43** with thalidomide as CRBN binder. Although **44** could successfully trigger BCL-6 degradation in a dose-dependent fashion, it showed a phenotypic profile similar to that of parent **43**. Despite achieving a sufficient cellular concentration, it failed to induce a significant anti-proliferative effect in diffuse large B-cell lymphoma cell lines. This was probably because a nuclear residual BCL6 level was detected. Although this work demonstrated for the first time that the macrocycle modality can be combined with PROTAC concepts, it clearly points out the challenges associated with the strategy.

Notably, Olsen et al. exploited macrocyclic peptides as POI ligands for developing epi-PROTACs.^127^ Epi-PROTACs have great potential as both pharmacological tools and therapeutics.^128^ In detail, the researchers exploited these chemotypes to develop the macrocycle-based PROTAC **45** (Figure 17), capable of selectively degrading class I HDACs 1–3 in cells, in a time- and concentration-dependent manner. **45** was based on the potent and class I-selective macrocyclic tetrapeptide inhibitor TpxB^Aoda^ as POI ligand,^129^ which was connected to thalidomide, by employing a modular “click chemistry” synthesis. Notably, this successful report shows that macrocyclic peptides can be elaborated into cell-permeable PROTACs.

Regarding approach (ii), it was superbly validated by A. Ciulli, one of the PROTAC pioneers.^130^ By harnessing the crystal structure of well-known **12** (Figure 17) in complex with the E3 ligase VHL and its target BRD4,^131^ the authors realized that a macrocyclic PROTAC could be designed to lock the conformation in the bound state. Particularly, macrocyclization was achieved by adding a cyclizing linker between the two ligand moieties of **12**, obtaining macroPROTAC-1 (**46**, Figure 17). The rational design was confirmed by the cocrystal structure of a **46**:VHL:BRD4 ternary complex. Despite a 12-fold loss in binary binding affinity for BRD4, **46** revealed cellular activity comparable to that of **12**, thus supporting macrocyclization as a successful strategy for PROTAC design. In this respect, to facilitate macrocyclization of PROTACs, a computational method has been proposed to automatically generate feasible cyclization by known chemical reactions.^124^ Moreover, this approach identifies attachment points, evaluates geometric compatibility, and ranks the resulting macrocyclic molecules by their predicted conformational stability with the target protein.^124^

Although in the reported examples the macrocycle is either a ligand or the linker and no extra functionality is added, we dubbed them as *multifunctional* PROTACS on the basis of the accomplished combination of two modalities.^27^

## 7 Combining PROTAC and Oligonucleotide Modalities

**Figure 18:**
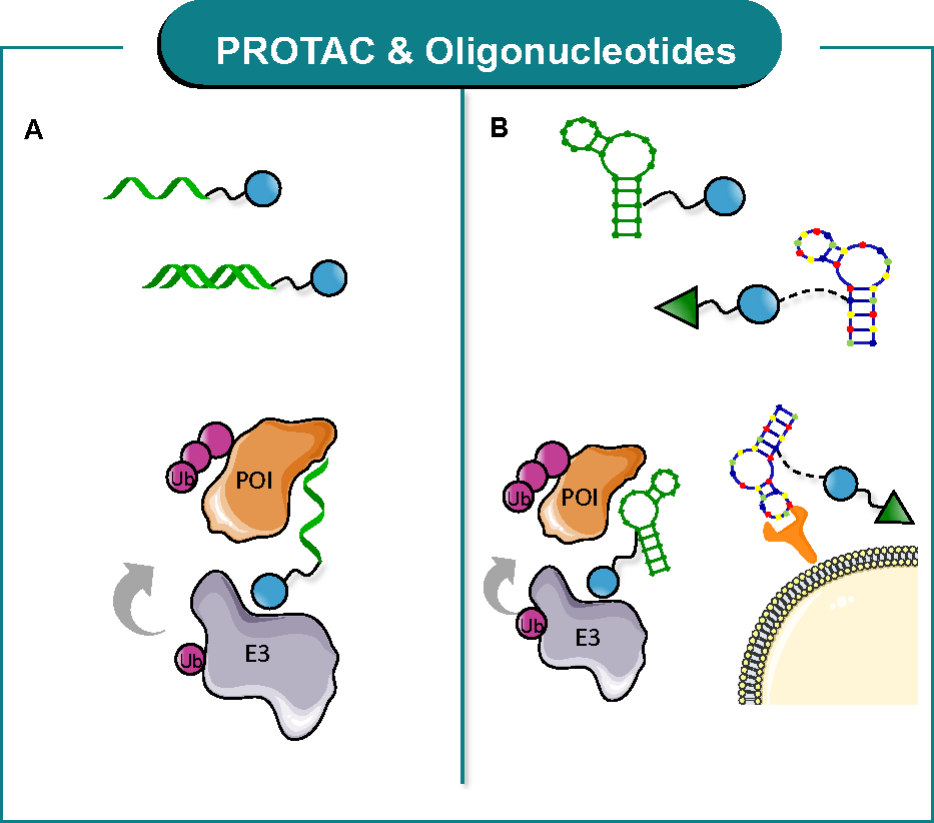
General structure and MoA of *oligonucleotide-based* PROTACs: (A) single-strand and double-strand oligonucleotide-based PROTACs and (B) aptamer-conjugate or aptamer-based PROTACs.

Oligonucleotide-based therapeutic modalities, such as ASOs, siRNA, microRNAs (miRNAs), and aptamers, are gaining new momentum in drug discovery.^52^ The majority of oligonucleotide modalities interact with a specific sequence of its target via complementary Watson–Crick base pairing, inhibiting gene expression. Hence, oligonucleotide therapeutics have a high selectivity, as they can be rationally designed based on the primary sequence of the target, allowing the modulation of patient-specific sequences for precision and personalized treatments.^132^ In the past decades, several oligonucleotide therapeutics have entered clinical trials, leading to the current approval of 11 oligonucleotide-based drugs across many disease areas.^133^ Notably, in 2017, the FDA and the European Medicines Agency approved nusinersen as the first ASO (i.e., short, single-stranded synthetic oligodeoxynucleotides) for the treatment of a neurological disease. Despite booming, oligonucleotide-based therapeutics suffer from poor drug-like properties and toxicity concerns. Potential liabilities may arise from their polyanionic nature, causing unexpected interactions with plasma and cellular proteins, unpredictable tissue accumulation, non-specific pro-inflammatory toxicities, and immune activation.^52^ Nevertheless, a number of chemical modifications and conjugation strategies aimed at improving nuclease resistance, binding affinity, and ADME-tox properties have been proposed over the years.^52^ In addition, oligonucleotide-based therapeutics have received ever-increasing attention for the potential to modulate “undruggable” targets that lack hydrophobic pockets and well-defined binding sites. Among them, TFs and RNA-binding proteins (RBPs) are essential for DNA repair, replication, transcription, and many RNA-dependent processes. When dysregulated, TFs and RBPs trigger numerous disease pathways. To address these undruggable POIs, PROTAC technology has successfully been combined with oligonucleotide-based therapeutic modalities, giving rise to innovative oligonucleotide-based PROTACs (Figure 18). In detail, two applications have been realized so far, namely (i) single-strand and double-strand oligonucleotide-based PROTACs (Figure 18A) and (ii) aptamer-based PROTACs (Figure 18B).

For the sake of clarity, we will solely focus on RNA-based PROTACs and the latest TF-targeting oligonucleotide-based PROTACs which have not been discussed in other recent reviews.^26,28^

**Figure 19:**
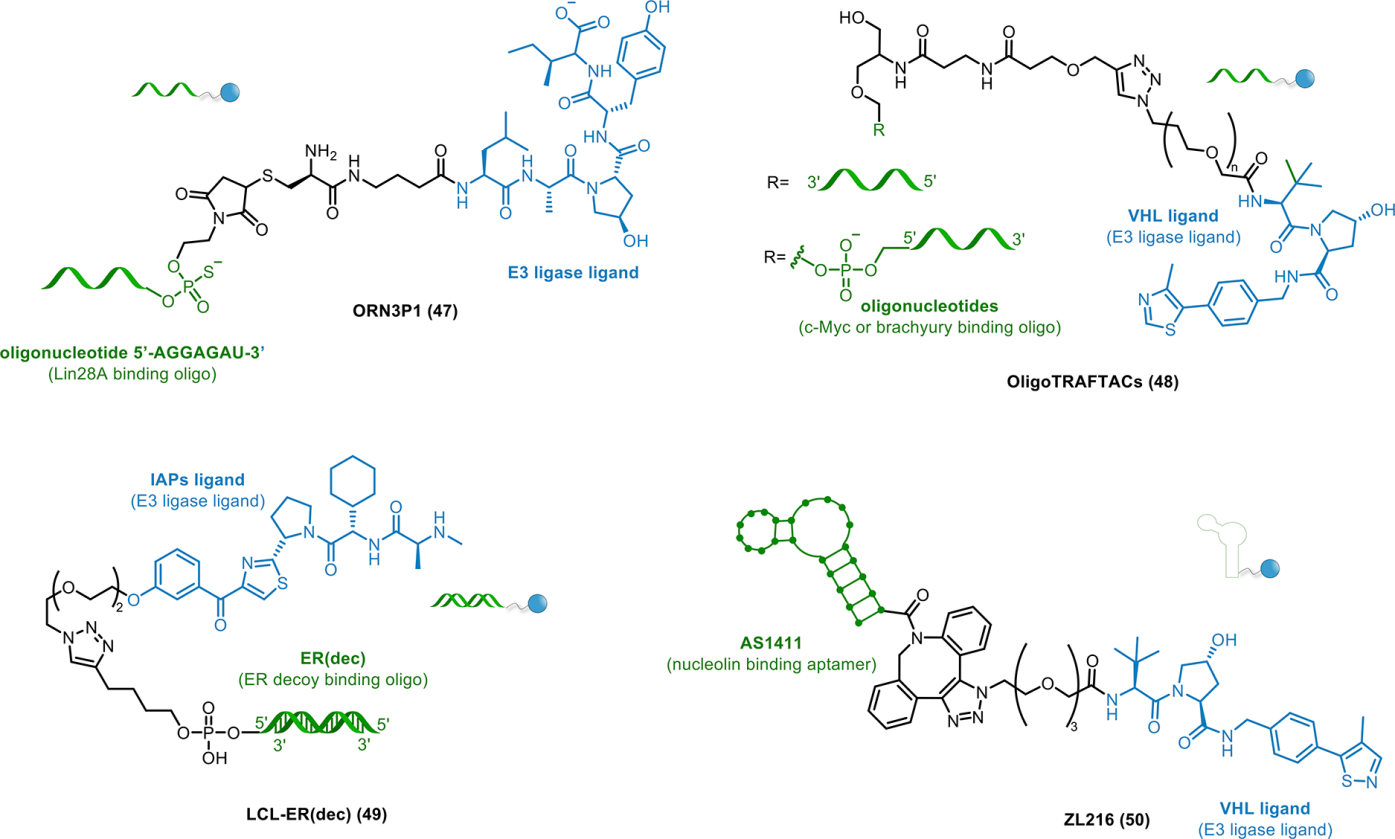
Design of oligonucleotide-based PROTACs.

Ghidini et al.^134^ developed the first degraders of RBPs (ORN3P1, **47**Figure 19) by employing—as RBP ligand—short oligonucleotides, designed from the RNA consensus binding element, linked to an E3 ligase-binding element. The oligonucleotide competes with native RNA for binding the RBP, and the simultaneous recruitment of E3 ligase allows target ubiquitination and degradation. By using a structure-based approach, a set of oligonucleotide analogues that selectively binds Lin28A, a stem cell factor and oncoprotein involved in several diseases, have been designed. Particularly, the authors structurally modified a short oligonucleotide, 5′-AGGAGAU-3′, which is sequence-identical with the native RNA-binding element of the RBP to enhance nuclease resistance, membrane permeability, and PK properties. A VHL-recruiting ligand was conjugated to the 5′-end of the oligonucleotide to provide **47**, which mediated target degradation in two cancer cell lines via the UPS. The RNA-PROTAC described in this recent publication truly expands the PROTAC concept by exploiting a short oligonucleotide as the POI ligand.

Starting from a chimeric DNA:CRISPR-RNA molecule, i.e., Transcription Factor TArgeting Chimera (TRAFTAC), that binds a dCas9-HaloTag7 fusion adaptor,^135^ Crews and co-workers^136^ described the development of the second-generation TRAFTACs, so-called “oligoTRAFTACs”. OligoTRAFTACs are constituted of a TF-binding oligonucleotide and an E3 ligase binder. As such, the oligonucleotide sequence recruits the transcription factor of interest (TOI), while the E3 ligase-binding element mediates cellular ubiquitination and subsequent degradation. A short oligonucleotide specific to c-Myc or T-box transcription factor (brachyury) as TOI was synthesized with a terminal alkyne at either the 3′ or 5′ end (**48**, Figure 19). With the alkyne-oligonucleotide in hands, a copper-catalyzed cycloaddition click reaction was then performed with an azide-containing VHL ligand, to afford **48** (Figure 19). Notably, oligoTRAFTACs mediate c-Myc and brachyury degradation in cell lines. Moreover, their *in vivo* applicability was demonstrated by using a zebrafish experimental model. This study clearly demonstrates that it is possible to develop a new class of rationally designed oligo-based degraders for extremely challenging targets, such as TFs.

Interestingly, a similar approach was recently exploited to develop PROTACs that degrade the estrogen receptor using decoy oligonucleotide ligands.^137^ Beside belonging to the nuclear hormone receptor superfamily, ERα can also act as a TF, forming transcriptional complexes on the DNA response sequence, thereby regulating gene expression. The developed decoy oligonucleotide is a double-stranded decoy, namely LCL-ER(dec) (**49**), designed from the sequence of the estrogen-responsive element that is known to tightly bind ERα. **49** has been functionalized with a terminal alkyne to be then conjugated to azide-bearing E3 ligase ligands, e.g., IAP ligand LCL16, thanks to a copper-catalyzed click reaction. Among the different subsets of synthesized degraders, **49** (Figure 19) showed the highest ERα degradation activity. This last piece of work gives evidence that the development of a PROTAC using a decoy ligand can be attractively applied for targeting TFs.

In parallel, the use of an aptamer to develop innovative PROTACs has been recently proposed (Figure 18B). Aptamers are single-stranded DNA or RNA oligonucleotides with a length less than 100, which are used as both POI ligand and targeted delivery agent.

Tan and co-workers^138^ exploited the aptamer AS1411 as the POI ligand of nucleolin, a protein highly expressed on the tumor cell surface and highly implicated in tumorigenesis and angiogenesis. Based on this, AS1411 was conjugated to VHL ligand via a dibenzylcyclooctyne copper-free click chemistry reaction, to give aptamer-based PROTAC ZL216 (**50**). Remarkably, **50** induced nucleolin degradation by UPS *in vitro* and *in vivo*. Furthermore, it also showed high selectivity for cancer cells in comparison to normal cells. This recent effort validates the combination of aptamer and PROTAC technologies and provides a promising strategy for the development of tumor-selective PROTACs.

Aptamers can be also used as targeted delivery units. In fact, they are often called “chemical antibodies” due to their function comparable to that of traditional Abs, but with numerous advantages.^139^ In fact, aptamers bind to their target with high specificity and affinity but, unlike Ab, possess unique characteristics, including easy synthesis, versatile chemical modification, and lack of immunogenicity.^140^

**Figure 20:**
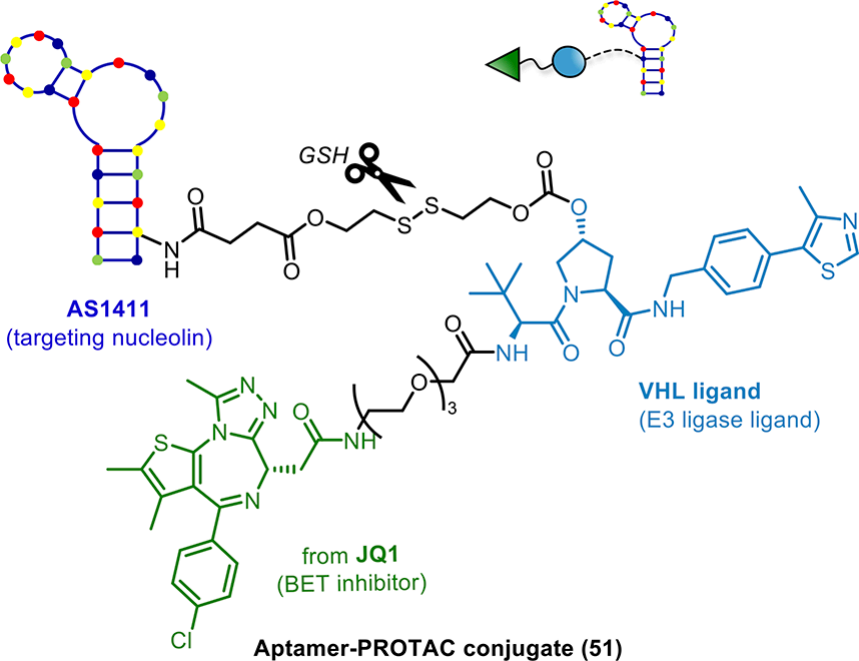
Design of aptamer-PROTAC conjugate **51**.

On this basis, Dong and Sheng developed the first aptamer–PROTAC conjugate (**51**) by combining the BET-targeting PROTAC **12** to AS1411 via a cleavable linker (Figure 20).^141^**51** showed a remarkable specificity for nucleolin-overexpressing MCF-7 breast cancer cells and a potent BET degradation effect. The advantages of AS1411 were highlighted by its excellent *in vivo* tumor targeting ability, reduced side effects on normal tissue, and improvement of drug-like properties, making this strategy highly appealing. Successfully, **51** not only combines two modalities in a single molecule but also is a truly *multifunctional* PROTAC, which harnesses the oligonucleotide aptamer as an effective targeting function.

## Conclusions

“The past is prologue”, as stated by Craig M. Crews, is the best description of the present-day PROTAC landscape.^26^ Given the potential of modulating currently undruggable targets, this new therapeutic modality has revolutionized small-molecule drug discovery paradigms. On the other hand, as PROTACs differ from established concepts of drug design, drug binding, and selectivity, a few key questions remain unsolved. Encouragingly, it seems that we are finding answer to these questions by developing *multifunctional* PROTACs:

- **Clinical translation.** The fundamental question—whether this new therapeutic modality will be used in clinical practice—might be figured out soon. The most advanced candidates, ARV110 (NCT03888612) and ARV471 (NCT04072952), have reached Phase I/II for the treatment of prostate and breast cancers, respectively. They have showed a favorable safety profile, tolerability, and anti-tumor activity, but long-term effects are largely unknown. Of note, ARV-471 is under evaluation in combination with palbociclib, whereas a trial of ARV-110 and abiraterone (NCT05177042) just started in January 2022. These combinations were all conceived based on the prototypical polypharmacology hypothesis that targeting two different nodes of cancer pathology may lead to superior efficacy, especially for highly drug-resistant forms. Thus, *multitarget* PROTACs might be powerful tools not only for the treatment of advanced resistant cancers but also for other complex diseases.
- **Modulable targets.** Another key issue is to expand the so-called PROTACtable genome,^142^ i.e., targets that could be modulated by PROTAC modality. In a recent analysis, Schneider et al.^142^ established ideal features and highlighted 95 different protein targets. However, so far, we have limited examples of protein target families, mainly belonging to nuclear receptors, bromodomains, and kinases. All these share similar features: They are soluble proteins with a nuclear or cytoplasmatic localization. Only a few examples have been reported of PROTACs targeting transmembrane proteins^143,144^ (two on GPCRs^145,146^ and none on ion channels), which are major drug targets in traditional drug discovery. The *oligonucleotide-based* PROTAC examples discussed herein illustrate that the PROTAC technology, when combined with this second modality, can be successfully applied to typical “undruggable” targets, such as TFs.
- **PROTAC design and physicochemical properties.** PROTAC drug design and development is predominantly an iterative process, with structure optimization guided mainly by chemical intuition, due to the limited availability of ternary complex 3D structures. Undoubtedly, such already challenging design is even more difficult when a PROTAC is combined with a second modality. First, the resulting PROTACs are chemically conjugated to a second framework, responsible for the second modality. Thus, the fact that each part retains the ability to interact with its specific target is an essential requirement. Second, conjugation may lead to PROTACs with unfavorable physicochemical properties such as MW and hydrophobicity increases. Integration of a second modality thorough a framework allowing structural overlapping (usually ring systems) may minimize such disadvantages. Similarly, incorporation of a photoswitchable unit into a PROTAC structure through a judicious azologization may afford *light-controllable* PROTACs with physicochemical properties comparable to those of parent PROTACs and simultaneous spatiotemporal control. On the other hand, the combination of PROTAC technology with *macrocycles* or *oligonucleotides*, both deemed to have poor drug-like properties, has yielded potent and effective degraders. Although, in principle, the combination with a second modality further complicates PROTAC design, the final outcome in terms of drug-likeness cannot be *a priori* established.
- **Selectivity.** A PROTAC does not always require selective binding to the POI, as is typically necessary for traditional small-molecule drugs. Owing to their MoA, PROTACs may prove to be more selective than the parent POI ligands. Although the phenomenon has yet to be completely explained, protein–protein interactions of the ternary complex may drive selectivity and potency. In fact, it has been shown that promiscuous inhibitors can be turned into selective degraders.^147^ Nevertheless, PROTACs may display toxic and unwanted side effects that can be removed or minimized by combining a second modality. *Light-controllable* PROTACs allow a fine control of degradation activity only upon irradiation, whereas targeting moieties of PROTAC *conjugates* allow selective delivery to target cells, with no toxicity to normal tissues. On the other hand, *macrocycle*- and *oligonucleotide*-based PROTACs recognize a POI with high specificity and selectivity, not only enhancing the degradation efficiency but also avoiding any off-target effects.
- **No clear SAR.** The need of identifying more systematic approaches to PROTAC development is of critical importance. Similar to what done by Corwin Hansch, father of QSAR, it might be helpful to develop a set of quantitative rules built on the knowledge of how a PROTAC molecule’s degradation activity correlates with its physicochemical properties. Clearly, in the case of PROTACs enriched with a second modality, additional considerations should be made, depending on the type of PROTAC we are dealing with. *Macrocycle*- and *oligonucleotide*-based PROTACs designed from novel chemotypes, covering a completely different chemical space, require a deeper understanding of how to manipulate their structure to optimize the activity.
- **Ternary complex.** The recognition process between a PROTAC and its protein targets is a multistep process owing to their high dynamicity and plasticity. This means that, to trigger POI degradation, a PROTAC should ensure a fast and precise recruitment of all players into the right shape and at the right time, across several steps. A further issue is the availability on the ternary complex of an accessible lysine that can be ubiquitinated. This, of course, calls for more reliable computational methods, which can allow straightforward structure-based drug design.^148^ In the past years, several *in silico* methodologies have been proposed to rationally model PROTAC-mediated ternary complexes, which have already demonstrated their utility in structure-based approaches. Clearly, this already complex scenario becomes even more challenging by the presence of a framework responsible for a second modality. Also, studying the formation of a ternary complex with POIs, like TFs and RBPs, which can produce homo- and hetero-multimeric complexes, is extremely complicated. While crystal structures of PROTAC-mediated ternary complexes continue to be reported, more precise computational modeling methodologies are urgently needed to rationally propose innovative PROTAC derivatives endowed with an additional activity.

The successful and unsuccessful efforts described herein clearly reflect the multiple challenges that medicinal chemists face in developing *multifunctional* PROTACs. The translation of academic drug discovery has been notoriously difficult, and this could be also the case for many of the academic innovations discussed herein. There is no easy recipe to follow, but a deeper and detailed understanding is required from many actors from many fields to fully exploit these opportunities and decisively enrich the PROTAC toolbox.

## Abbreviations

Ab: antibody
ACNP: antibody conjugate nanoparticle
ADC: antibody–drug conjugate
BCL6: B cell lymphoma 6
cIAP1: inhibitor of apoptosis protein
CLL1: C-type lectin-like molecule-1
CRBN: cereblon
DAR: drug–antibody ratio
DEACM: diethylaminocoumarin
DMNB: 4,5-dimethoxy-2-nitrobenzyl
ERRα: estrogen-related receptor α
ERα: estrogen receptor α
FOLR1: folate α receptor
GSH: glutathione
HALG: hypoxia-activated leaving group
HER2: human epidermal growth factor receptor 2
mAb: monoclonal antibody
MDM2: mouse double minute 2 homologue
MEK1/2: mitogen-activated protein kinase
miRNA: microRNA
MTDL: multitarget-directed ligand
NPOM: 6-nitropiperonyloxymethyl
NTR: nitroreductase
PARP: poly(ADP-ribose) polymerase
P-mPD: PROTAC-mediated protein degradation
POI: protein of interest
PROTAC: proteolysis targeting chimera
RBP: RNA-binding protein
siRNA: small interfering RNA
SMDC: small molecule–drug conjugate
TF: transcription factor
TOI: transcription factor of interest
TRAFTAC: transcription factor targeting chimera
UPS: ubiquitin–proteasome system
VHL: von Hippel Lindau
